# Targeted eDNA Survey Enables Consistent Detection of the Invasive Blue Crab in Trans‐Border Adriatic Coastal‐Marine Ecosystems

**DOI:** 10.1002/ece3.74462

**Published:** 2026-10-12

**Authors:** Maria Dolores De Donno, Francesco Zangaro, Pietro Arnosti, Sara Bergamin, Josip Boban, Maria Pia Bozzetti, Armando Cazzetta, Cristiano De Pittà, Dario Dessanai, Filippo Drigo, Olumide Temitope Julius, Laura Ključe, Jelena Lušić, Francesca Marcucci, Roberto Massaro, Valeria Morelli, Isabella Moro, Nikša Nazlić, Michele Pellizzato, Marco Rainò, Mahallelah Shauer, Marta Tasinazzo, Emanuele Troli, Valeria Specchia, Maurizio Pinna

**Affiliations:** ^1^ Department of Biological and Environmental Sciences and Technologies University of Salento Lecce Italy; ^2^ Research Centre for Fisheries and Aquaculture of Acquatina di Frigole, DiSTeBA University of Salento Lecce Italy; ^3^ Department of Biology University of Padova Padova Italy; ^4^ Eduforma Srl Padova Italy; ^5^ Public Institution for the Management of Protected Natural Areas of Split‐Dalmatia County Split Croatia; ^6^ Public Institution for the Management of Protected Natural Areas of Dubrovnik‐Neretva County Dubrovnik Croatia; ^7^ Institute of Oceanography and Fisheries Split Croatia; ^8^ Blu Marine Service Soc Coop San Benedetto del Tronto Italy

**Keywords:** COI metabarcoding, eDNA, Mediterranean marine ecosystems, species‐specific primers

## Abstract

The invasive blue crab *Callinectes sapidus* poses a significant ecological and economic threat to Mediterranean marine ecosystems, highlighting the need for sensitive and reliable tools for its early detection. We developed and validated a species‐specific environmental DNA (eDNA) assay designed for sensitive detection of *C. sapidus* . Species‐specific primers targeting a 464 bp fragment of the mitochondrial COI gene were designed, and their specificity was confirmed through in silico analyses using Primer‐BLAST and Sanger sequencing, and their effectiveness was validated via high‐throughput sequencing (HTS) of environmental DNA (eDNA) samples. The assay based on eDNA proved to be reliable and robust in confirming the presence of blue crab DNA in five cross‐border Adriatic pilot areas impacted by this invasive species. In addition, the dominant OTU was consistently present in all sites, while haplotype network analysis using ASVs revealed a shared central haplotype across Adriatic populations, supporting their genetic cohesiveness and a common invasion history. These results emphasize the importance of molecular tools for tracking invasion dynamics and reinforce the potential for uniform genetic profiles to facilitate management efforts. Overall, our findings demonstrate that targeted eDNA approaches are essential for effective early detection and containment strategies in marine ecosystems threatened by biological invasions, and that the blue crab in the Adriatic Sea constitutes a potential genetically cohesive invasive entity.

## Graphical abstract

In this research we developed an experimental assay for the identification of the invasive blue crab and applied it to the detection through targeted eDNA and NGS in marine‐coastal ecosystems of the Adriatic Sea. We demonstrated the feasibility of monitoring this species' presence using eDNA analysis, and our results indicate a genetic homogeneity among the blue crab populations in the Adriatic Sea.

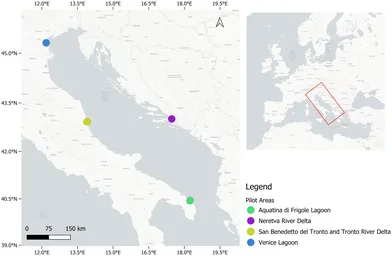

## 1 Introduction

Human activities are the dominant drivers of marine non‐indigenous species (NIS), defined as organisms occurring outside their native distribution ranges. Shipping represents the primary introduction pathway, accounting for 51.2% of new records via ballast water discharge and hull fouling (Marine Non‐Indigenous Species in Europe's Seas 2024). The corridor pathway contributes a further 14.3% of introductions, particularly through man‐made waterways connecting previously isolated biogeographic regions, such as the Lessepsian migration event leading to the influx of Red Sea species into the Mediterranean Sea through the Suez Canal (Marine Non‐Indigenous Species in Europe's Seas 2024; Khalil et al. 2025).

Aquaculture constitutes an additional pathway, facilitating NIS spread through both the escape of farmed non‐native species and the unintentional transfer of associated organisms (Rius et al. 2011).

Following their introduction, ecological factors such as the lack of top‐down population control, trophic flexibility, and reproductive strategies can enable a subset of NIS to undergo niche shifts and establishment. This leads to niche overlap, competitive displacement of native species, ecosystem alteration, and biodiversity loss (Soberón and Nakamura 2009; Peterson et al. 2011; Guisan et al. 2014). These impacts occur in the context of multiple co‐occurring anthropogenic pressures, including pollution, habitat degradation, and climate change (Specchia et al. 2022; Flitcroft et al. 2025).

A well‐documented example of a successful marine invader is the Atlantic blue crab, *Callinectes sapidus* (Figure 1), a species native to the western Atlantic Ocean and most likely introduced into the Mediterranean Sea during the 1940s via ballast water discharge (Nehring 2011). Since its introduction, its populations have expanded rapidly across a wide range of Mediterranean coastal habitats, with a marked acceleration observed since approximately 2017, resulting in substantial predation pressure on native biota (Castriota et al. 2024; Gavioli et al. 2026). This species is an opportunistic predator, exploiting a broad spectrum of prey, including fish, invertebrates, polychaetes, macrophytes and, in particular, bivalves. Such trophic plasticity enhances its ecological impact and may contribute to documented declines in bivalve populations across invaded areas, from oysters in the Ebro Delta (Spain) to the aquaculture of *Ruditapes philippinarum* in the Po Delta (Italy). In this and other areas, the species has become the dominant member of benthic communities, coinciding with significant declines in native crab populations (Belgrad and Griffen 2016; Brundu et al. 2025; Gaglio et al. 2025). In addition, *C. sapidus* exhibits meaningful physiological tolerance, being a euryhaline species capable of withstanding a wide range of salinity and temperature conditions. This adaptability enables it to colonize diverse environments, including coastal waters, estuaries, and lagoons (Castriota et al. 2024; Shauer et al. 2025). Its invasion success is further supported by r‐selected life‐history traits, with females producing up to 4 million eggs per spawning event and exhibiting flexible reproductive phenology, with reproductive peaks varying along latitudinal and geographic gradients (Prager et al. 1990; Turner et al. 2003). The combination of ecological plasticity, opportunistic feeding behavior, and high reproductive output underpins its rapid spread and substantial impact on Mediterranean coastal and marine ecosystems.

**FIGURE 1:**
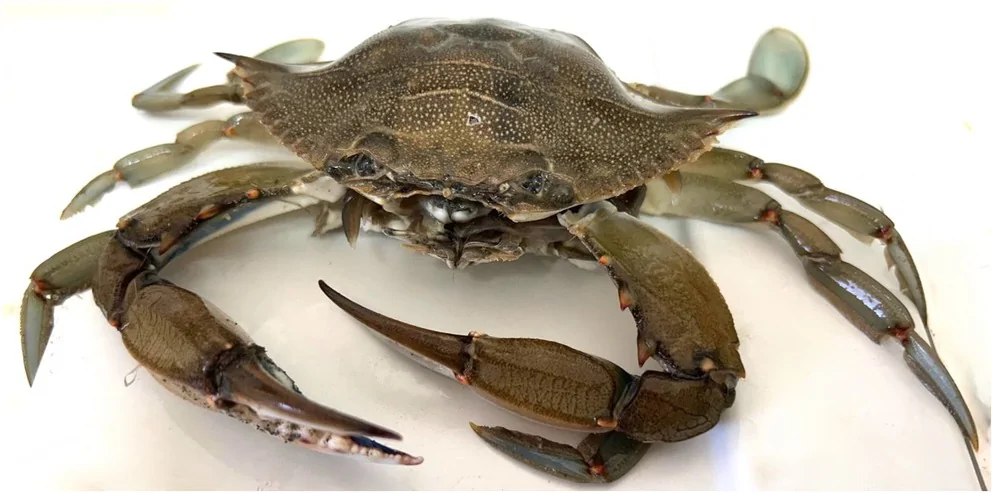
Adult specimen of *Callinectes sapidus* . Photograph by the authors.

The presence of *C. sapidus* is frequently associated with reduced native biodiversity and altered trophic structures, underscoring the importance of early detection and continuous monitoring. According to Regulation (EU) No 1143/2014, early detection is defined as the confirmation of an invasive alien species before it becomes widely established, and effective management relies on prevention, early detection, and rapid response (Genovesi et al. 2015). Despite its ecological impact, *Callinectes sapidus* is not currently included in the European Union list of invasive alien species of Union concern. Recent long‐term analyses of its invasion dynamics across the Adriatic, Ionian, and central Mediterranean subregions suggest that its expansion may be facilitated by increasing sea surface temperatures. These findings highlight the urgent need for coordinated management actions to mitigate the emerging ecological and socio‐economic risks associated with this species. In this context, inclusion of *C. sapidus* in the EU list could support harmonized surveillance, standardized impact assessment, and timely intervention across Member States, ultimately contributing to the development of an effective early‐warning framework (Shauer et al. 2025).

Conventional survey methods (e.g., trapping and visual census) frequently fail to detect low‐density invasions. For instance, in the Ebro Delta, a lag of approximately 5 years was observed between the first detection of the blue crab and the onset of exponential population growth. This limitation can be overcome by the development and adoption of molecular tools for biodiversity monitoring; among these, environmental DNA (eDNA) metabarcoding. By extracting DNA directly from environmental samples such as water or sediment, amplifying universal genetic markers such as Cytochrome c oxidase I (COI), 18S rRNA, or 12S rRNA using degenerate primers, and applying high‐throughput sequencing, metabarcoding enables the simultaneous identification of entire biological communities. This approach can represent a rapid, reliable, and cost‐effective early‐warning system, particularly well‐suited for detecting non‐indigenous species at very low abundances. Thereby, it may deliver timely alerts that are critical for effective management and rapid response strategies, as demonstrated to date in a few studies (Specchia et al. 2022; Zangaro et al. 2024).

However, despite its broad applicability, the effectiveness of this approach is often constrained by incomplete reference databases, which can limit taxonomic resolution and lead to ambiguous or unassigned sequences. This issue is particularly relevant in marine and transitional ecosystems, where gaps in barcode libraries remain significant (Porter and Hajibabaei 2018; Hestetun et al. 2020; Zangaro et al. 2021).

In addition, metabarcoding relies on universal degenerate primers that simultaneously amplify DNA from multiple taxa. As a result, DNA from the target species competes with DNA from all co‐occurring organisms during PCR amplification. Consequently, species occurring at low abundances may be underrepresented in the sequencing output or excluded during bioinformatic filtering, potentially reducing the sensitivity of metabarcoding for the early detection of rare or invasive species.

In parallel, species‐specific eDNA assays based on targeted PCR amplification have been applied and validated as effective tools for the early detection of invasive and rare species. These approaches rely on highly specific primers that selectively amplify DNA from a single target species directly from environmental samples, enabling reliable detection even at very low DNA concentrations and population densities (Ficetola et al. 2008; Zangaro et al. 2024; Liang et al. 2026). Compared to metabarcoding, targeted assays offer higher sensitivity and are particularly suited for monitoring high‐risk species, although their performance depends on rigorous primer design to avoid cross‐amplification with non‐target taxa.

In this study, we build on these advances by developing species‐specific primers targeting Mediterranean populations of *Callinectes sapidus* , representing the first targeted eDNA assay for this species, and applying them to screen pilot sites for early evidence of its presence. Primer specificity was initially assessed through in silico analyses and subsequently validated in vitro and in situ using both Sanger sequencing and high‐throughput sequencing (HTS) of eDNA samples. This approach enables detection of *C. sapidus* DNA, providing an effective early‐warning system for blue crab incursions and supporting timely management actions aimed at protecting Mediterranean marine biodiversity.

## 2 Materials and Methods

### 2.1 Design and Validation In Silico of Primers Targeting the Blue Crab

In order to develop a useful tool for targeted detection of the invasive species *Callinectes sapidus* , the first step was the design of a pair of primers suitable for amplifying a segment of the COI gene of this species.

For this purpose, we downloaded from the NCBI GenBank database (https://www.ncbi.nlm.nih.gov/genbank) five COI sequences of *C. sapidus* representative of Mediterranean populations.

To amplify the variable region flanked by conserved regions, the position of the forward primer was first established using Primer‐BLAST. To minimize potential cross‐reactivity of the designed forward primer with non‐target species, a list of approximately 1300 aquatic animal species inhabiting transitional and coastal ecosystems co‐occurring with *Callinectes sapidus* in the Mediterranean Sea was constructed. For these species, sequences of the “Folmer region” of the COI gene were searched and eventually retrieved from the NCBI GenBank database, resulting in a subset of approximately 600 species used for testing (Table S1). The forward primer was tested in silico on the retrieved Folmer COI sequences to evaluate potential mismatch, annealing, and amplification efficiency. Subsequently, candidate reverse primers suitable for our purpose were manually designed, outside the Folmer region, by inspecting the multiple sequence alignment of five sequences obtained with Clustal Omega, approximately 500 bp downstream of the forward primer position. This distance was selected to obtain an amplicon of about 500 bp, which represents an optimal length for both Sanger sequencing and NGS analyses, while ensuring reliable species‐level identification. The specificity of the selected primer pair was further assessed using Primer‐BLAST against the entire NCBI nucleotide database. Only the primer pair predicted to exclusively amplify *Callinectes sapidus* , without amplification of non‐target sequences, was retained for subsequent analyses.

### 2.2 DNA Extraction From Blue Crab Tissue

For DNA extraction, approximately 200 mg of *Callinectes sapidus* muscle tissue was dried at 37°C for about 20 h and then thoroughly homogenized using a PowerLyzer instrument (QIAGEN, Germany).

Genomic DNA was extracted from the tissue using the DNeasy PowerSoil Pro Kit (QIAGEN, Germany), following the manufacturer's protocol with the addition of a proteinase K incubation step to enhance lysis efficiency. The DNA was stored at −20°C immediately after extraction.

### 2.3 Primer Validation on Tissue by PCR and Sanger Sequencing

The species‐specific primers were synthesized with the support of Invitrogen, Waltham, MA, USA. The melting temperature, the probability of secondary structure generation, and the possibility of self‐annealing into primer dimers were evaluated using the freely available web‐based tool “Multiple Primer Analyzer” software of Thermo Fisher Scientific (Thermo Fisher Multiple Primer Analyzer). The optimal annealing temperature was empirically determined through a series of PCR optimization experiments. Annealing temperatures ranging from 48°C to 60°C, in 2°C increments, were evaluated under otherwise identical reaction conditions. The final annealing temperature of 54°C was selected because, using the same initial concentration of DNA extracted from crab muscle tissue, it produced the strongest and most reproducible amplification.

PCR reactions were carried out in a total volume of 50 μL, containing 5 μL of 10× reaction buffer, 1 μL MgCl_2_ (50 mM), 1 μL dNTP mix (10 mM), 1 μL forward primer (10 μM), 1 μL reverse primer (10 μM), 10 ng of genomic DNA, 0.2 μL Platinum Taq DNA polymerase (5 U/μL; Life Technologies, Carlsbad, CA, USA), and sterile water up to the final volume. The concentration of genomic DNA extracted from animal tissue was measured using a Qubit 2.0 Fluorometer (Thermo Fisher Scientific, Waltham, MA, USA), and 10 ng of genomic DNA was used as a template for each PCR reaction.

The thermal cycling conditions were as follows: initial denaturation at 95°C for 5 min; 30 cycles of denaturation at 95°C for 30 s, annealing at 54°C for 30 s, and extension at 72°C for 1 min; followed by a final extension at 72°C for 10 min. PCR products were separated on a 1% agarose gel and visualized under UV light.

The amplicon was purified using the EuroSAP PCR Enzymatic Clean‐up Kit (EuroClone, Italy), following the manufacturer's instructions, and subsequently Sanger‐sequenced by BMR Genomics (Padova, Italy). The resulting sequences were labeled as CrabForSpecific and deposited in GenBank of NCBI.

### 2.4 Water Sampling and Study Areas

For each replicate, 1 L of surface water was collected in sterile containers to minimize the risk of contamination. Although *C. sapidus* is a benthic species, surface water sampling was considered appropriate in shallow transitional environments, where hydrodynamic processes and sediment resuspension may facilitate eDNA dispersal throughout the water column. Following collection, samples were immediately transported to the genetics laboratory of the Department of Biological and Environmental Sciences and Technologies (DiSTeBA, University of Salento, Lecce, Italy) for subsequent processing. When immediate transport was not possible, samples were stored at 4°C for a maximum of 24 h and shipped in refrigerated boxes to preserve the integrity of the genetic material. Water sampling was conducted in the following areas (Figure 2).

**FIGURE 2:**
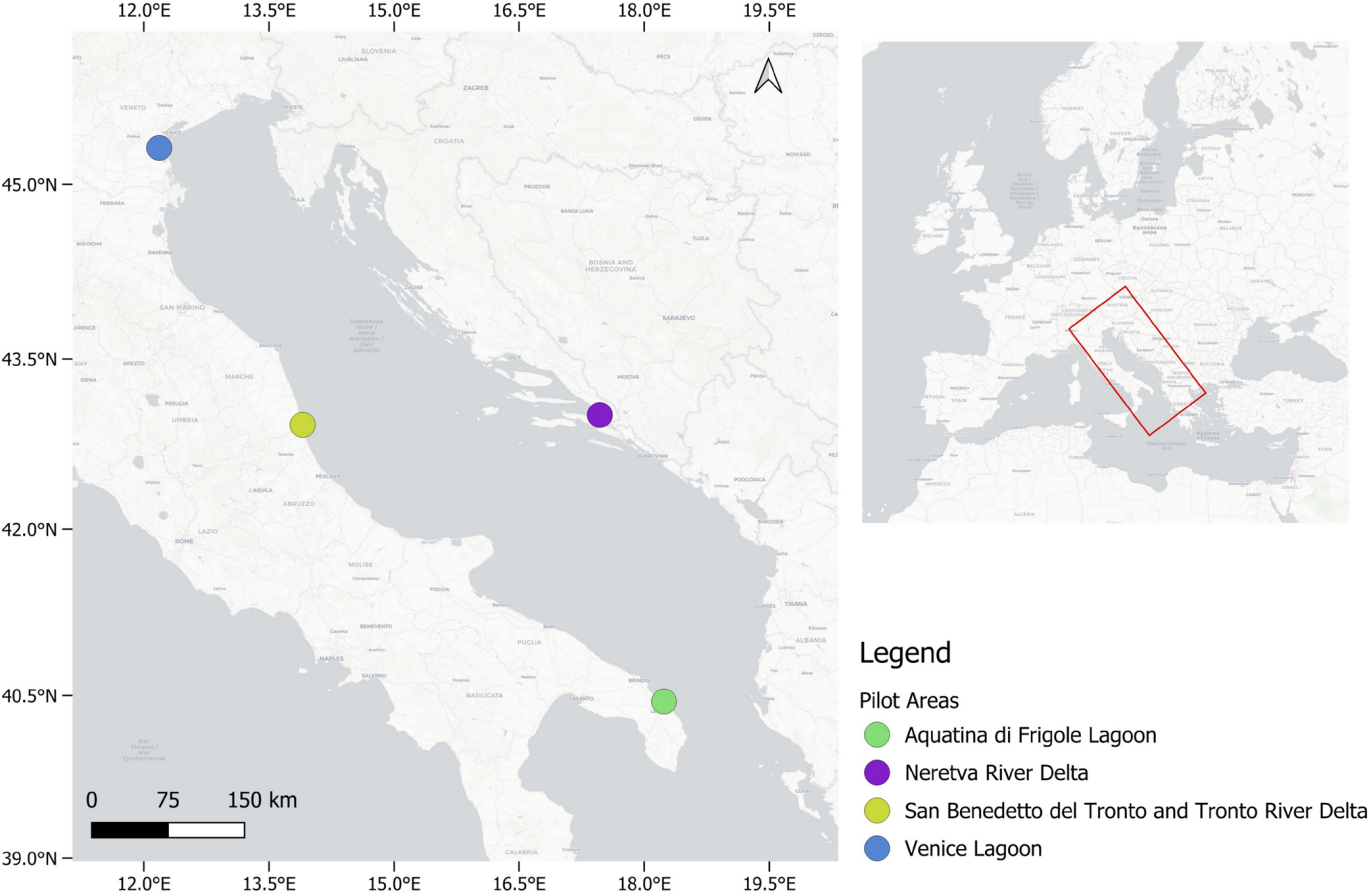
Map of the study area indicating the location of the pilot sampling sites across the investigated regions. The map was generated using QGIS v3.28 (https://qgis.org).

#### 2.4.1 Aquatina di Frigole Lagoon (Italy)—Three Sampling Sites

The first study area was the coastal lagoon of Aquatina di Frigole, included within the NATURA 2000 Site “Aquatina di Frigole” (IT9150003), located along the Adriatic coast of the Salento peninsula (Apulia Region, southeastern Italy). The site hosts the Aquatina di Frigole Research Centre for Fisheries and Aquaculture of the University of Salento. The lagoon covers approximately 42 ha and extends for about 2 km, with a maximum depth of 1.5 m and a maximum tidal excursion of approximately 34 cm. It is directly connected to the Adriatic Sea through a channel about 400 m long and 15 m wide on its southern boundary, while freshwater input is provided on the northern side by a branch of the Giammatteo canal.

#### 2.4.2 Neretva River Delta (Croatia)—One Sampling Site

The second study area was the Neretva River. The Neretva River basin extends over approximately 5580 km^2^, with its upper course in Bosnia and Herzegovina and its lower estuary and delta located in Croatia. The Neretva represents the only river in Croatia forming a true delta at its mouth, covering an area of about 196 km^2^. This estuarine system is highly productive and biologically diverse. However, it has been meaningfully impacted by human activities. The remaining wetlands and lagoons host important ornithological and ichthyological communities and are protected under the NATURA 2000 Network as well as national legislation.

#### 2.4.3 San Benedetto del Tronto Coast and Tronto River Delta (Italy)—Three Sampling Sites

The third study area was located along the central Adriatic coast, between the port of San Benedetto del Tronto and the Tronto River mouth, approximately 4 km south of the city. This coastal stretch is characterized by strong interactions between natural processes and anthropogenic pressures. It includes the Special Area of Conservation (SAC) “Litorale di Porto d'Ascoli” (IT5340001), which extends over 109 ha within the Sentina Regional Nature Reserve. The reserve is managed by the Municipality of San Benedetto del Tronto in collaboration with regional and provincial authorities. La Sentina is included in the NATURA 2000 Network as a ZSC (IT5340001).

#### 2.4.4 Venice Lagoon (Italy)—One Sampling Site

The last study area was located in the Venice Lagoon. The Venice Lagoon, the largest transitional ecosystem in the Mediterranean basin, is a Natura 2000 site located in the northern Adriatic Sea, spanning approximately 550 km^2^. It is subdivided into three main hydrographic basins, each connected to the sea through distinct inlets: the northern basin (Lido inlet), the central basin, which includes the city of Venice and the industrial area of Marghera (Malamocco inlet), and the southern basin (Chioggia inlet), which includes the town of Chioggia.

### 2.5 eDNA Extraction

A total of 1 L of surface water was collected per replicate. Triplicate samples were collected at Aquatina, the San Benedetto del Tronto coast, and the Neretva River Delta, while duplicate samples were collected at the Venice Lagoon and the Tronto River Delta, resulting in a total of 22 eDNA samples.

Before filtering water samples, a prefiltration step was implemented. This process was necessary to prevent filter clogging and minimize the presence of Polymerase Chain Reaction (PCR) inhibitors. Specifically, each 1‐L replicate was pre‐filtered through a 0.5 mm conical sieve mesh to retain suspended particulate material. Each 1‐L replicate was then filtered through a 0.45 μm cellulose filter of 47 mm diameter (Advantec Mixed Cellulose Ester filters). Following this, each filter was singularly used in the DNA extraction process using the DNeasy PowerWater kit (QIAGEN, Germany) following the manufacturer's protocol. To improve DNA extraction efficiency, the lysis step was conducted on a Tissuelyser III (Qiagen, Germany) at 30 Hz for 5 min. The extracted DNA was immediately stored at −20°C.

### 2.6 Primer Validation on Environmental Samples by PCR and NGS

The species‐specific primers CSapFor (5′‐GCGACCCTGTTCTCTACCAAC‐3′) and CSapRev (5′‐AGCACCCATGGATAGAACGT‐3′) were used to amplify eDNA samples collected from the different pilot areas.

PCR reactions were carried out in a total volume of 50 μL, containing 5 μL of 10× reaction buffer, 1 μL MgCl_2_ (50 mM), 1 μL dNTP mix (10 mM), 1 μL forward primer (10 μM), 1 μL reverse primer (10 μM), 10 ng of total eDNA extract, 0.2 μL Platinum Taq DNA polymerase (5 U/μL; Life Technologies, Carlsbad, CA, USA), and sterile water up to the final volume. The concentration of total DNA in each eDNA extract was measured using a Qubit 2.0 Fluorometer (Thermo Fisher Scientific, Waltham, MA, USA), and 10 ng of total extracted DNA was used as PCR template. As the concentration of target DNA cannot be determined in environmental samples prior to amplification, template input was standardized based on total eDNA concentration to ensure consistent PCR performance. Since the objective of this study was target detection rather than quantification, no normalization of target DNA was performed.

The thermal cycling conditions were as follows: initial denaturation at 95°C for 5 min; 30 cycles of denaturation at 95°C for 30 s, annealing at 54°C for 30 s, and extension at 72°C for 1 min; followed by a final extension at 72°C for 10 min.

All PCR products were purified with a PureLink PCR purification kit (Invitrogen, Carlsbad, CA, USA), quantified by a Qubit 2.0 Fluorometer (Invitrogen, Carlsbad, CA), and submitted to IGATech (Udine, Italy) for library preparation and sequencing. Amplicons were purified and subjected to library preparation using the DNA‐Seq library prep kit (Tecan, Männedorf, Switzerland). The fragmentation step was not applied. Libraries were sequenced on the AVITI platform (Element Biosciences, San Diego, CA) in 300‐bp paired‐end mode. High‐throughput sequencing of the COI amplicons generated 10,773,406 raw reads, averaging about 500,000 raw reads per sample. Raw reads were processed using an internal bioinformatics pipeline. Paired‐end reads were merged using FLASH v1.2.11 when overlap was sufficient, while non‐overlapping reads were retained. Primer sequences were removed using Cutadapt v2.7, and low‐quality bases were trimmed. Quality‐filtered reads were then processed within the QIIME v1.9.1 framework, including chimaera detection using VSEARCH v2.14.1 and OTU clustering using an open‐reference approach. Taxonomic assignment was performed using a curated COI reference database based on GenBank sequences and the RDP classifier, applying a minimum identity threshold of 97% and a confidence threshold of 0.50. Only OTUs meeting these criteria were retained for downstream analyses. In parallel, Amplicon Sequence Variants (ASVs) were generated for high‐resolution phylogenetic inference by applying a 100% sequence identity threshold.

### 2.7 Multiple Sequence Alignment and Phylogenetic Inference

Multiple sequence alignment was performed using the algorithm MAFFT v7.526 (Katoh and Standley 2013), executed via Python 3. To improve the phylogenetic tree readability and interpretation, the 2526 obtained *Callinectes sapidus* ASVs were filtered by relative abundance prior to multiple sequence alignment and phylogenetic inference. Read counts for each ASV were expressed as a percentage of total reads across the entire ASV matrix, and only ASVs exceeding a threshold of 0.1% were retained, reducing the initial set of 2526 ASVs to a reduced set of 96 candidate sequences spanning the four investigated pilot areas. The multiple sequence alignment was performed on 124 COI sequences comprising 96 *C. sapidus* ASVs obtained through metabarcoding, together with additional reference sequences belonging to other *Callinectes* species and a *Scylla serrata* sequence used as an outgroup.

Reference sequences for phylogenetic inference were programmatically retrieved from the NCBI GenBank nucleotide database using a Python script built on Biopython's Entrez module (Cock et al. 2009). For the target taxon, *Callinectes sapidus* COI sequences between 500 and 2000 bp were queried, and a random subsample of candidate records was downloaded and parsed. From these, 24 reference sequences were retained, selected to simultaneously maximize fragment overlap and geographic representation by sampling preferentially across distinct reported areas of origin. Congeneric reference sequences were obtained by querying the same marker gene within the genus *Callinectes*, excluding *C. sapidus* records. Again, only sequences sharing the target region were retained for the multiple sequence alignment and successive phylogenetic inference. An outgroup sequence was selected by first determining the taxonomic family of *C. sapidus* via the NCBI Taxonomy database, then querying for COI sequences within the same family but outside the genus *Callinectes*. The closest well‐annotated relative covering the same amplicon fragment, *Scylla serrata* , was retained.

Multiple sequence alignment was carried out under the ‐‐auto strategy with gap‐opening and gap‐extension penalties (‐‐op 3.0, ‐‐ep 0.5) set to limit extensive indel placements and preserve reading frame integrity. Alignment dimensions (sequence number and alignment length) were confirmed programmatically using Biopython's AlignIO module (Cock et al. 2009). The alignment was then visually inspected and trimmed at both ends to a uniform length before phylogenetic inference.

Maximum likelihood phylogenetic inference was performed in IQ‐TREE v3.1.3 (Wong et al. 2026) under a GTR + G + I substitution model, with automatic thread‐count optimization, which benchmarked one to three threads and selected two as optimal for this dataset. The best‐fit model was automatically selected by ModelFinder. Tree search began from 98 initial parsimony trees, from which the 20 highest‐likelihood trees were retained as the candidate tree set and refined via IQ‐TREE's stochastic nearest‐neighbor‐interchange hill‐climbing algorithm. Sequences identical across the alignment were computationally collapsed for the search and reinserted into the final tree. The search continued until the ultrafast bootstrap (UFBoot) split‐frequency correlation coefficient stabilized (final value = 0.993), completing after 260 iterations (best log‐likelihood = −1771.464). Branch support was assessed with 1000 UFBoot pseudo‐replicates (Hoang et al. 2018). Nodes with UFBoot support ≥ 95% were considered well‐supported. The resulting maximum‐likelihood tree was exported in the Treefile format and visualized and annotated in iTOL v7 (Letunic and Bork 2024).

### 2.8 Haplotype Network Analysis

To evaluate the genealogic relationships and spatial genetic structuring among the most abundant *C. sapidus* haplotypes across the species' native range, the Mediterranean Sea, and the investigated Adriatic Sea pilot areas, a *C. sapidus* haplotype network analysis was conducted. The same dataset of 96 *C. sapidus* ASVs and 24 *C. sapidus* reference sequences was analyzed, resulting in a combined dataset of 120 sequences spanning 16 localities of origin (Aquatina di Frigole Lagoon, Neretva River Delta, San Benedetto del Tronto and Tronto River Delta, Venice Lagoon, Mississippi, Maryland, Texas, Florida, Connecticut, Louisiana, Jamaica, Spain, Brazil, Gulf of Cadiz, Venezuela, and Mexico).

The 96 ASVs and 24 reference sequences were extracted as a subset of the multiple sequence alignment described for the phylogenetic inference, restricted to the *C. sapidus* reference sequences, so that the haplotype network and the maximum likelihood tree share an identical alignment coordinate system. Sequence data and locality metadata were compiled into a single NEXUS file, with each sequence annotated by locality of origin as a categorical trait. Gap positions were treated as missing data, according to the PopArt v 1.7 default handling of indels (Leigh and Bryant 2015).

Haplotype network reconstruction was performed in PopArt v 1.7 (Leigh and Bryant 2015) using the TCS statistical parsimony algorithm (Templeton et al. 1992; Clement et al. 2000) under the default 95% parsimony connection limit, which estimates the maximum number of mutational steps between haplotypes that can be considered non‐homoplasious with 95% confidence. Geographic origin was mapped onto network nodes as a categorical trait to visualize the spatial distribution of haplotypes across the native range, and the Mediterranean Sea and the investigated Adriatic Sea pilot areas representing the invaded range.

## 3 Results

### 3.1 Design and Validation In Silico of Primers Specific for the Blue Crab

The selected primer pair was subsequently evaluated using Primer‐BLAST against the entire NCBI nucleotide database. This analysis confirmed the amplification of *C. sapidus* , with no predicted amplification of non‐target species. The sequences and names of the selected primers are as follows:

*CSapFor*: GCGACCCTGTTCTCTACCAAC;

*CSapRev*: AGCACCCATGGATAGAACGT.

The position of the primer pair relative to the reference sequence of the target species is shown in Figure 3.

**FIGURE 3:**
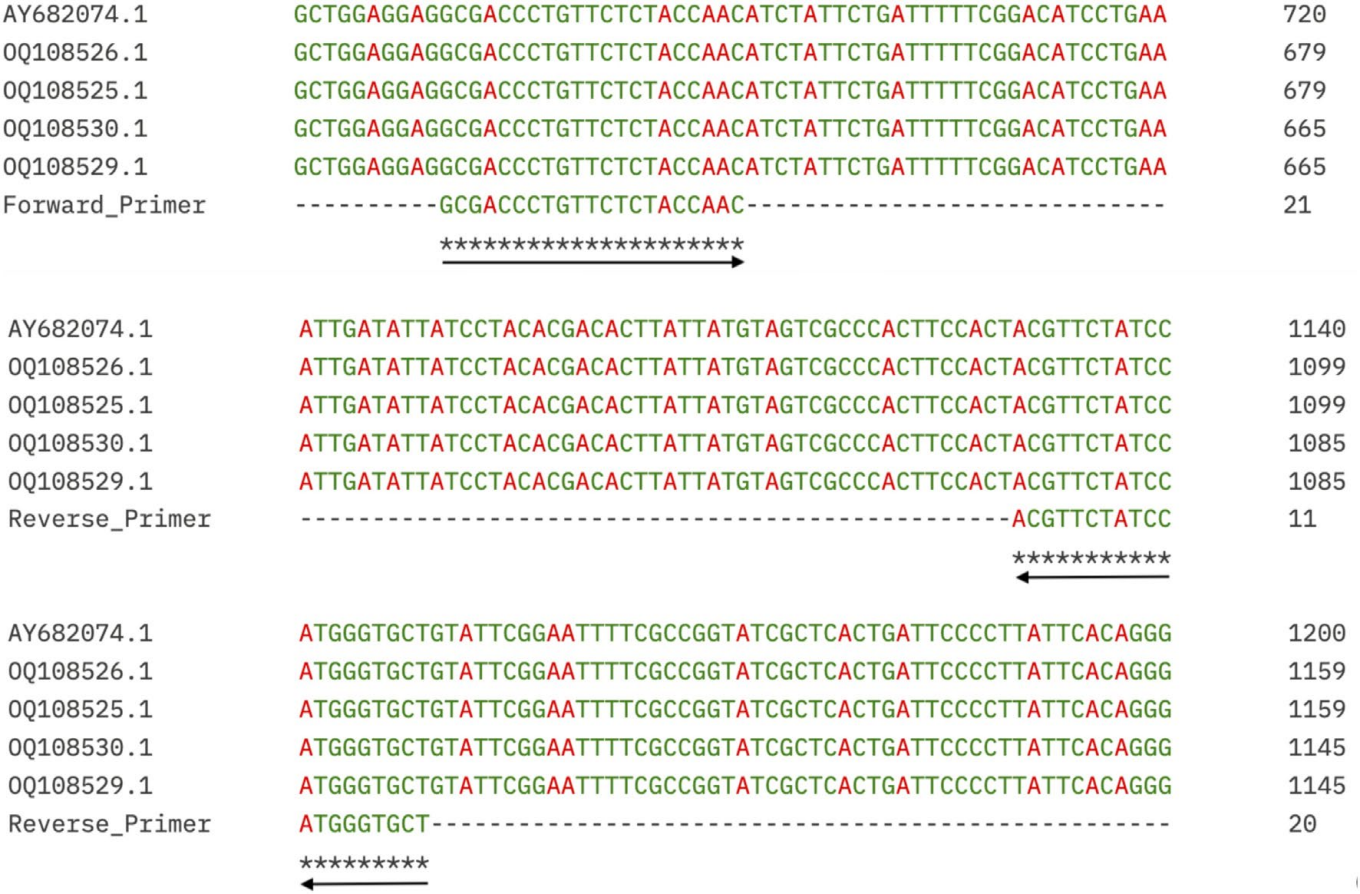
Multiple sequence alignment of the five *Callinectes sapidus* COI sequences used for primer design, showing the positions of the forward (*CSapFor*) and reverse (*CSapRev*) primers relative to the reference sequence.

### 3.2 Validation of Species‐Specific Primers on DNA Extracted From Blue Crab Tissue by Using Sanger Sequencing

To experimentally validate the efficiency and specificity of the designed primer pair, genomic DNA was extracted from the muscle tissue of one *Callinectes sapidus* individual and, as controls, from one individual of *Penaeus aztecus* and one of *Mytilus galloprovincialis* . PCR amplifications were performed using the *CSapFor/CSapRev* primer pair on DNA extracted from tissue samples.

These amplifications generated a single amplicon of the expected size (464 bp) from *Callinectes sapidus* tissue DNA, with no visible bands detected under the tested PCR conditions in reactions performed on DNA from the two non‐target control species (Figure 4).

**FIGURE 4:**
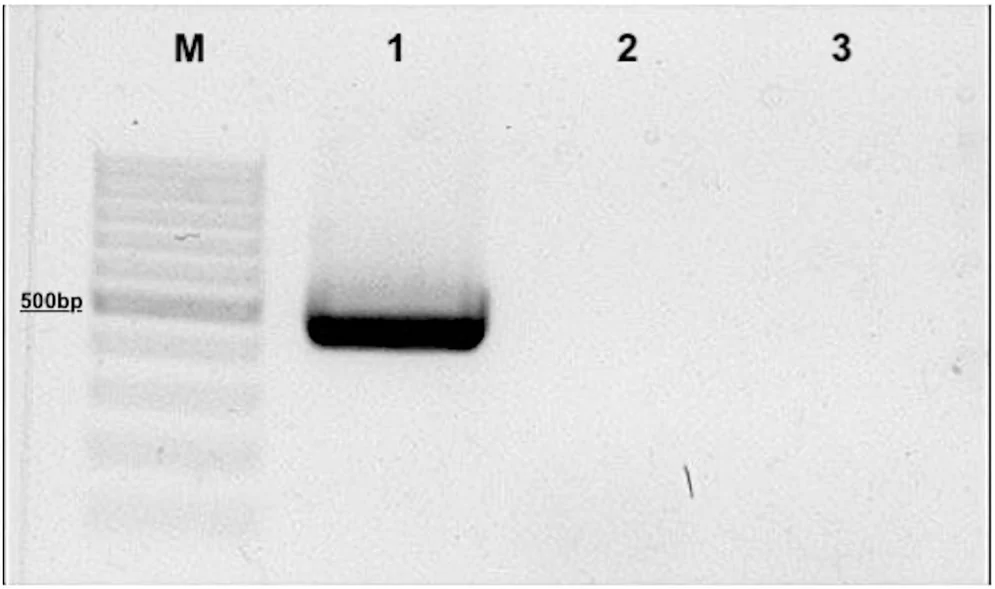
Agarose gel electrophoresis (1% agarose, 90 V, 20 min in TAE) of *C. sapidus* species‐specific primers products. Lane M: DNA marker GeneRuler 100 bp DNA Ladder (Thermo Fisher Scientific, Waltham, MA); lane 1: *Callinectes sapidus* ; lane 2: *Penaeus aztecus* ; lane 3: *Mytilus galloprovincialis* . Stained with SYBR Safe DNA Gel Stain (Thermo Fisher Scientific, Waltham, MA) and visualized under UV light.

To further validate the specificity of the amplification, the PCR product was subjected to Sanger sequencing. The resulting sequence (CrabForSpecific) was queried against the NCBI BLAST database, showing a match exclusively with *Callinectes sapidus* sequences, with 99% query coverage, an *E*‐value of 0.0%, and 100% sequence identity.

These results confirm that the amplified fragment corresponds to the intended target region. The absence of visible amplification products in the tested non‐target species, together with the in silico evaluation performed against a broad range of available sequences, supports the specificity of the designed primer pair.

### 3.3 Validation of Species‐Specific Primers on Environmental DNA by Using NGS

To evaluate the suitability of the species‐specific primer pair for the sensitive detection of the invasive species from environmental DNA, the performance of the species‐specific primer pair CSapFor/CSapRev was tested on eDNA samples using high‐throughput sequencing (HTS) of obtained amplicons. PCR amplifications were conducted on eDNA collected from the selected pilot sites, for a total of 22 eDNA samples, and the resulting amplicons were subjected to high‐throughput sequencing. A total of 10,773,406 reads were obtained, with an average of approximately 500,000 reads per sample.

The results of the NGS analysis showed that all OTUs assigned at the species level were identified as *Callinectes sapidus* or were taxonomically affiliated with this species. The remaining OTUs were either unassigned or classified at higher taxonomic levels. In total, 68 OTUs were taxonomically attributed to *C. sapidus* . Among these, the most abundant OTU in terms of read counts (KU987676) was detected across all pilot areas (Figure 5), further supporting the specificity and robustness of the primer pair in complex environmental samples. The presence of *C. sapidus* DNA was detected in all sampling sites, demonstrating the ability of the assay to identify the species even at low DNA concentration.

**FIGURE 5:**
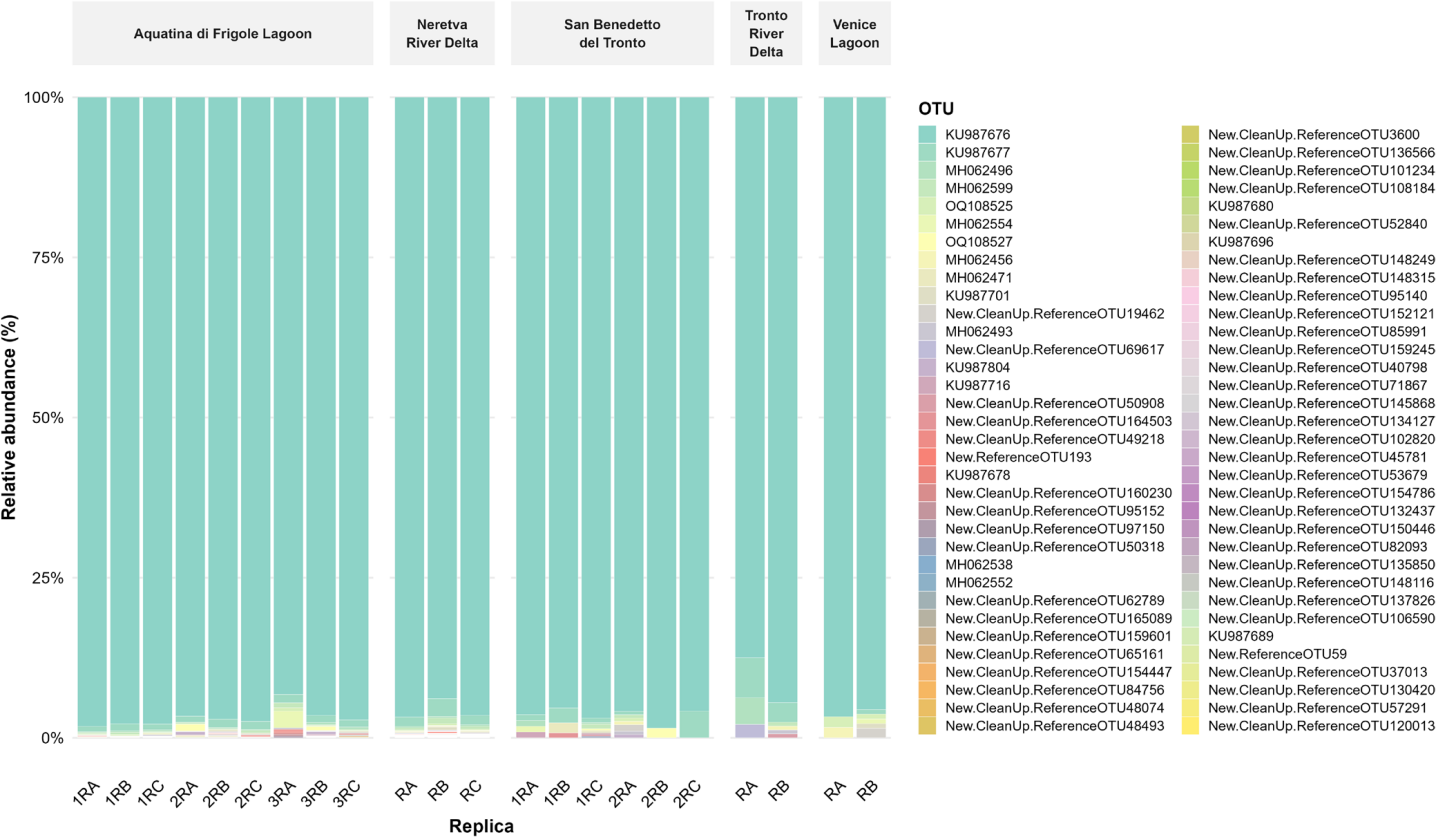
Relative abundance (%) of *Callinectes sapidus* ‐associated OTUs across all eDNA samples, shown per replicate and sampling area. Sampling areas are indicated above each panel, while individual replicates are shown along the *x*‐axis. The OTU KU987676 was the most abundant and consistently detected across all pilot areas, while the remaining OTUs showed lower relative abundances. All the reported OTUs were generated in the present study.

### 3.4 Phylogenetic Validation of Metabarcoding Annotations

To independently validate the taxonomic annotations generated by the bioinformatic pipeline following eDNA metabarcoding, a phylogenetic inference analysis, based on a Maximum Likelihood algorithm, was conducted using 96 ASVs of *Callinectes sapidus* together with 27 reference COI sequences representing the phylogenetic diversity of the genus *Callinectes*, and one *Scylla serrata* sequence as an outgroup (Figure S1). The topology deduced from phylogenetic inference allowed the identification of two well‐supported lineages among the reference taxa, consistent with previously conducted molecular phylogenies for the genus *Callinectes*. A first group, called “*bocourti*”‐group, and a second group, called “*danae*”‐group, were thus coherently identified (Robles et al. 2007).

The observed support is consistently high at the species and terminal cluster levels. ASVs derived from eDNA metabarcoding analysis show consistent clustering with the *C. sapidus* reference sequences and remain clearly distinct from other congeneric species included in the analysis. The inclusion of *S. serrata* also provides a stable external root, confirming the separation between the genus *Callinectes* and the outgroup lineage.

The large number of ASVs included in the analysis showed that branch lengths within the *C. sapidus* clade are generally very short, with internal support values showing a median UFBoot of approximately 50% across the entire tree, indicating limited sequence divergence between the identified COI variants.

Overall, the phylogenetic reconstruction demonstrated that all metabarcoding‐derived ASVs occupy an evolutionarily coherent position within the *C. sapidus* lineage, clearly separated by a broad and phylogenetically structured representation of congeneric diversity, independently validating taxonomic annotations obtained through OTU‐ and ASV‐based pipelines.

### 3.5 Blue Crab Haplotype Network

To elucidate the phylogenetic relationships and the spatial structuring of the dominant *Callinectes sapidus* variants across the species' native range, the Mediterranean Sea, and the Adriatic Sea pilot areas, a haplotype network analysis was conducted. The TCS‐based haplotype network built on 96 *C. sapidus* ASVs and 24 *C. sapidus* reference sequences resolved a star‐like topology anchored by a predominant central hub, with short branch lengths of 1 to 6 mutational steps connected via intermediate median vectors (Figure 6).

**FIGURE 6:**
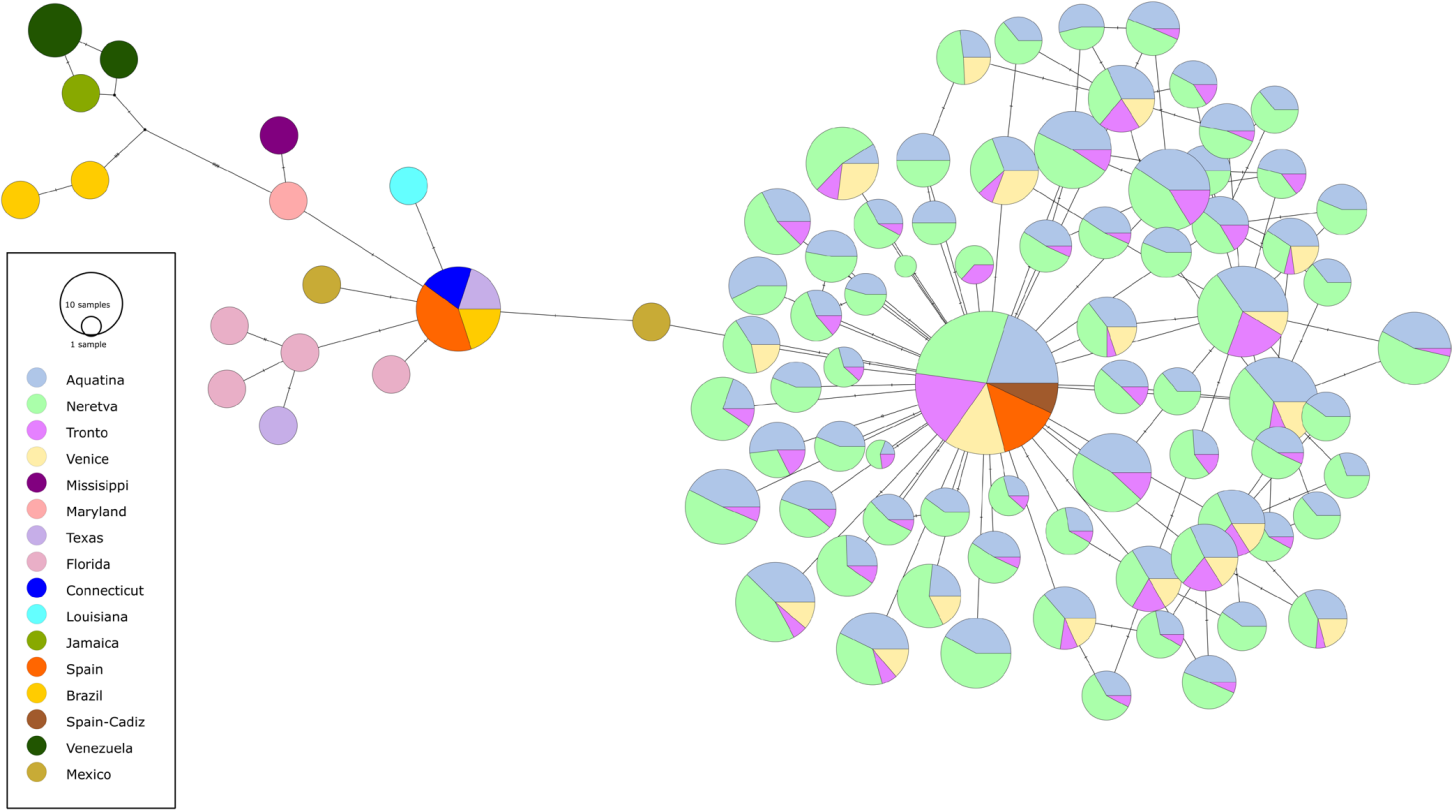
TCS‐based *Callinectes sapidus* Haplotype Network built on 96 *C. sapidus* ASVs spanning four Adriatic Sea pilot areas (Aquatina di Frigole Lagoon, Neretva River Delta, San Benedetto del Tronto, and Tronto River Delta, Venice Lagoon) and 24 *C. sapidus* reference sequences spanning 12 localities of origin (Mississippi, Maryland, Texas, Florida, Connecticut, Louisiana, Jamaica, Spain, Brazil, Gulf of Cadiz, Venezuela, and Mexico).

The Adriatic Sea samples formed a complex clustering exhibiting a star‐like network topology, characterized by a highly frequent central hub haplotype surrounded by multiple single‐mutation tip‐haplotypes, alongside minor secondary sub‐clusters reflecting localized in situ diversification. While the primary haplotype was ubiquitously distributed across all Adriatic Sea pilot areas and Mediterranean Sea reference sites, distinct spatial frequency partitioning was observed, marked by high haplotype richness in the Neretva River Delta and Aquatina di Frigole Lagoon compared to a subset of variants apparently more localized in the Venice Lagoon.

The dominant Mediterranean hub appears directly connected to reference sequences from Mexico and sequentially to the broader Atlantic/Mediterranean secondary hub, supporting the hypothesis of a shared invasion history across Mediterranean sub‐basins and suggesting evidence in accordance with the hypothesis of the species' introduction from the Gulf of Mexico via ballast water transport (Shauer et al. 2025; Shauer, Massaro, et al. 2026; Shauer, Rainò, et al. 2026). Native Western Atlantic reference sequences formed geographically structured peripheral lineages connecting to the primary Mediterranean cluster across short mutational distances via intermediate median vectors, with South American variants exhibiting the greatest relative evolutionary divergence.

## 4 Discussion and Conclusions

The research presented in this manuscript has culminated in the development and thorough validation of a novel molecular tool that holds significant value for both the scientific community and environmental monitoring professionals. This tool is designed to facilitate the early detection and continuous surveillance of the invasive alien species, the blue crab *Callinectes sapidus*, which has increasingly become a concern in marine ecosystems. Historically, the blue crab has been recognized as a species of considerable ecological and economic importance in various marine environments, particularly along the Atlantic coasts of North America and the Gulf of Mexico (Morgan et al. 1996; Nehring 2011). However, its recent appearance and proliferation in the Adriatic Sea have raised alarms due to observed shifts in local populations and the potential impacts associated with climate change and global warming trends. The rising number of sightings and the increasing abundance of this species in the region serve as clear indicators of broader ecological transformations driven by environmental changes, which threaten the stability of native communities and ecosystems (Shauer et al. 2025, Shauer, Massaro, et al. 2026; Shauer, Rainò, et al. 2026).

The presence of the blue crab presents an escalating challenge for mollusk aquaculture enterprises operating along the Adriatic coast, as the species can significantly impact economically valuable shellfish populations. Moreover, the ecological implications of this invasion are complex and multifaceted. As a generalist predator, the blue crab has the potential to exert substantial predation pressure on local benthic communities, possibly disrupting existing trophic relationships and reducing biodiversity (Shauer et al. 2025, Shauer, Massaro, et al. 2026; Shauer, Rainò, et al. 2026). The full extent of its ecological impacts on natural aquatic ecosystems remains under investigation, with ongoing studies aiming to elucidate these effects in detail. A critical aspect of advancing such research is the ability to detect the presence of the species at an early stage of invasion, which can meaningfully improve management and mitigation efforts (Woodell et al. 2021).

Approaches based on environmental DNA (eDNA) are becoming increasingly central to biodiversity assessment and ecological monitoring of Mediterranean aquatic ecosystems, complementing traditional morphological and functional ecological research methodologies, particularly in coastal and marine ecosystems. In particular, studies of Mediterranean lagoons and coastal systems have established a solid ecological foundation, ranging from the hierarchical distribution of biodiversity at different spatial scales in lagoon ecosystems (Basset et al. 2008), to the resistance and resilience of ecosystem descriptors to dystrophic and hypoxic crises (Basset et al. 2013), to the dynamics of detritus processing in transitional and fluvial environments (Fonnesu et al. 2004; Sangiorgio et al. 2004), and to the population biology of key benthic indicator species (Barbone et al. 2007). These systems are further shaped by complex hydrogeological processes (Margiotta et al. 2021) and continue to host species of extreme conservation interest (Marrocco et al. 2018; Zangaro et al. 2020). Starting from this ecological context, DNA‐based tools, including barcoding and metabarcoding, have progressively expanded biomonitoring capacity in several taxonomic groups, from benthic macroinvertebrates (Tzafesta et al. 2021; Pinna et al. 2021, 2024) to phytoplankton communities (Tzafesta et al. 2022; Specchia et al. 2023; Zangaro et al. 2025), while specific gap analyses in DNA barcode reference libraries have highlighted both the potential and current limitations of these approaches in Mediterranean aquatic ecosystems (Specchia et al. 2020; Pinna et al. 2021; Tzafesta et al. 2022). More recently, the integration of artificial intelligence and machine learning tools has further expanded the range of analytical tools available for ecological monitoring of transitional waters (Cazzetta et al. 2026), while species distribution modeling and fish community studies have supported the adaptive management of both exploited and native fish populations, from basin‐scale fishing patterns in the Adriatic Sea (Shauer, Massaro, et al. 2026) to aging fish assemblages in tropical waters (Julius et al. 2026). Together, these studies illustrate a well‐established and evolving suite of molecular and ecological tools for biodiversity monitoring in Mediterranean and transitional water ecosystems, providing the methodological and conceptual foundations on which this study builds to address the specific challenge of early detection of the invasive blue crab *Callinectes sapidus* .

In this context, our study introduces a molecular detection tool based on environmental DNA (eDNA) analysis. This approach allows for species identification through the analysis of DNA fragments shed into the environment by living organisms, obtained from minimal water samples. The core of this method relies on the design of species‐specific primers. These primers were carefully designed and validated through both computational analyses and experimental testing. Their effectiveness was further demonstrated through next‐generation sequencing (NGS) analysis of COI gene amplicons derived from eDNA samples. Water samples for this study were collected from multiple pilot areas along both coasts of the Adriatic Sea, all of which have documented occurrences of the blue crab.

Compared with universal COI primer pairs commonly employed in eDNA metabarcoding, the species‐specific primers developed in this study are intended for targeted detection of *Callinectes sapidus* . Universal degenerate primers, such as mCOIintF/dgHCO2198, are effective for amplifying DNA from a broad range of taxa and are therefore widely used for biodiversity assessments. Furthermore, during amplification with universal primers, DNA from the target species competes with DNA from all co‐occurring organisms present in the sample. Consequently, when the target species occurs at low abundance, its sequences may be underrepresented in the sequencing output or removed during bioinformatic filtering, potentially reducing detection sensitivity. In contrast, the species‐specific eDNA assay developed in this study provides enhanced sensitivity for the detection of *C. sapidus* . Indeed, previous analyses of the same Adriatic pilot areas using the widely adopted degenerate primer pair mCOIintF/dgHCO2198 did not consistently detect *C. sapidus* , whereas the species‐specific primers developed here successfully identified the species across all investigated sites.

Although taxonomic annotation in eDNA metabarcoding is typically performed using Bayesian classifiers trained on curated reference databases, this similarity‐based classification alone cannot explicitly assess the evolutionary coherence of identified sequences. In this context, phylogenetic inference was employed as an independent validation step to evaluate whether the complete set of ASVs identified in environmental samples was evolutionarily consistent with *Callinectes sapidus* . The resulting topology clearly showed that all metabarcoding‐derived ASVs are located in the *C. sapidus* lineage while remaining separated from closely related representative species. This validation provides solid independent support to confirm the robustness of the taxonomic annotations produced by the bioinformatic pipeline.

The reference dataset was specifically designed to include a phylogenetic representation of the genus *Callinectes* consistent with those independently reported for the genus *Callinectes*, also using different mitochondrial markers (Robles et al. 2007). Accordingly, the obtained results indicate that multiple sequence alignment and subsequent phylogenetic inference produced a valid and reproducible phylogenetic tree. The approach demonstrates that the identified ASVs occupy the expected phylogenetic position with respect to the reference sequences, providing an additional validation step that targeted primer design, combined with eDNA metabarcoding, can represent an extremely efficient and reliable tool for detecting the presence of non‐native species even in the very early stages of invasion (Shauer et al. 2025; Shauer, Rainò, et al. 2026). Furthermore, the results obtained demonstrate that the clustering of over 2500 ASVs should not be interpreted as evidence of extensive evolutionary diversification within the sampled populations. Amplicon Sequence Variants maintain nucleotide differences at a single‐base resolution, thus allowing even the smallest sequence polymorphisms to be preserved. Consequently, numerous ASVs may represent closely related mitochondrial haplotypes that differ only by one or a few nucleotide substitutions. This interpretation is supported by the short branch lengths and internal support generally observed within the *C. sapidus* clade, indicating that the detected molecular diversity largely reflects subtle intraspecific variations.

To deeply evaluate the phylogenetic relationships and geographic structuring among the most abundant *C. sapidus* haplotypes across the species' native range, the Mediterranean Sea, and the investigated Adriatic Sea pilot areas, a *C. sapidus* haplotype network analysis was conducted, as previously done in studies using eDNA metabarcoding results for phylogeographical analyses (Turon et al. 2020, 2025). The results revealed a star‐shaped network topology, dominated by a primary ancestral hub comprising sequences from the investigated Adriatic areas and sequences from Mediterranean populations, showing complex genetic divergence consisting of numerous short mutational distances. This topology suggests an invasion scenario in which a primary introduction, particularly from the Gulf of Mexico, may have been accompanied by secondary or multiple introduction events, supported by the direct connection between the predominant Mediterranean haplotype and sequences native to Mexico and Texas, followed by a rapid post‐settlement demographic expansion (Nei et al. 1975; Roman and Darling 2007; Specchia et al. 2017; Zangaro et al. 2024; Shauer et al. 2025, Shauer, Massaro, et al. 2026; Shauer, Rainò, et al. 2026). The broad distribution of the primary haplotype across all Adriatic locations also reflects high gene flow and potential panmixing, driven by prolonged pelagic larval drift and regional hydrodynamics (Epifanio et al. 1984), facilitated by the strong oceanographic connectivity characteristic of the Adriatic Sea. Together with this widespread expansion, the identification of low‐frequency, single‐mutation terminal haplotypes, as well as localized secondary clusters, suggests the possibility of ongoing in situ microdiversification after the initial settlement phase. The observed spatial partitioning suggests, in fact, the existence of localized demographic processes, likely due to localized environmental filters. Furthermore, while the direct linkage to Mexican lineages strongly implicates maritime vectors (e.g., ballast water transport) from the Gulf of Mexico as the primary invasion route, the peripheral placement of minor western Atlantic variants, separated by short mutational intervals, underscores the importance of continuous high‐density sampling in both native and invaded areas to further refine fine‐scale connectivity patterns.

These results open the possibility of broader applications of this innovative and highly efficient tool in coastal and marine ecosystems of the Mediterranean Sea. The approach not only allows for the early detection of non‐native species in the initial stages of invasion (Shauer et al. 2025; Shauer, Rainò, et al. 2026; Zangaro et al. 2024), but also for the collection of additional data on their genetic diversity, allowing for the reconstruction of invasion dynamics at the molecular level through the identification of potentially unique haplotypes originating in the species' native range or following their introduction into the Mediterranean Sea. By integrating phylogenetic inference and haplotype network analyses with high‐resolution ASVs clustering, this work provides a robust framework for confirming taxonomic identity and increases the reliability of ecological inferences derived from eDNA‐based datasets. This strategy is particularly effective for both invasive and cryptic taxa, in which species discrimination is essential for accurate biodiversity assessments and robust ecological monitoring.

To date, this study represents one of the few experimental demonstrations confirming the feasibility of detecting an invasive species through targeted eDNA analysis. Previous research validated a probe‐based qPCR method for detecting the invasive blue crab from eDNA, highlighting the usefulness of eDNA probe‐based qPCR for monitoring invasive species (Delrieu‐Trottin et al. 2025). Our study demonstrates that using species‐specific primers and next‐generation sequencing (NGS) enables effective detection and phylogenetic and haplotype analyses of *Callinectes sapidus* , complementing eDNA qPCR‐based methods.

The results proved to be highly robust across five distinct pilot areas, each characterized by different ecological conditions, as well as across various sampling sites within each area, and in biological replicates per site. Importantly, the operational taxonomic unit (OTU) classified as *C. sapidus* , which exhibited the highest sequence read abundance, was consistently identified across all sampled regions, providing strong evidence for the reliability and sensitivity of the assay.

The findings from this research support the use of the developed molecular toolkit as a rapid and reliable method for species identification in environmental samples. The robustness of the results indicates that the primers pair developed in this study improves the sensitivity of *C. sapidus* detection in environmental samples compared with broad‐range metabarcoding approaches, supporting early detection and ongoing monitoring efforts. This approach offers a practical advantage for management and research, enabling timely responses to invasion events and informing control strategies.

In conclusion, the proliferation of the blue crab in the Adriatic Sea exemplifies the complex interactions between biological invasions and climate change. Environmental shifts, such as rising temperatures and changing oceanic conditions, are likely to continue favoring the establishment and spread of this invasive species, underscoring the urgent need for comprehensive monitoring and management strategies. Employing molecular tools like eDNA analysis provides a powerful means to detect and track invasive species, even in the absence of physical specimens. Such techniques not only enhance early detection capabilities but also offer insights into genetic diversity, invasion pathways, and the origins of invasive populations. This genetic information is crucial for developing targeted management and control measures, ultimately helping to protect native ecosystems and mitigate ecological and socio‐economic impacts associated with invasions. Overall, integrating molecular detection methods into invasive species management represents a vital step toward more effective and timely responses to biological invasions in a changing world.

## Author Contributions

**Maria Dolores De Donno:** data curation (supporting), formal analysis (supporting), investigation (supporting), methodology (supporting), writing – original draft (supporting), writing – review and editing (supporting). **Francesco Zangaro:** data curation (supporting), formal analysis (supporting), investigation (supporting), methodology (supporting), writing – original draft (supporting), writing – review and editing (supporting). **Pietro Arnosti:** writing – review and editing (equal). **Sara Bergamin:** writing – review and editing (equal). **Josip Boban:** writing – review and editing (equal). **Maria Pia Bozzetti:** writing – review and editing (equal). **Armando Cazzetta:** writing – review and editing (equal). **Cristiano De Pittà:** writing – review and editing (equal). **Dario Dessanai:** writing – review and editing (equal). **Filippo Drigo:** writing – review and editing (equal). **Olumide Temitope Julius:** writing – review and editing (equal). **Laura Ključe:** writing – review and editing (equal). **Jelena Lušić:** writing – review and editing (equal). **Francesca Marcucci:** writing – review and editing (equal). **Roberto Massaro:** writing – review and editing (equal). **Valeria Morelli:** writing – review and editing (equal). **Isabella Moro:** writing – review and editing (equal). **Nikša Nazlić:** writing – review and editing (equal). **Michele Pellizzato:** writing – review and editing (equal). **Marco Rainò:** writing – review and editing (equal). **Mahallelah Shauer:** writing – review and editing (equal). **Marta Tasinazzo:** writing – review and editing (equal). **Emanuele Troli:** writing – review and editing (equal). **Valeria Specchia:** conceptualization (lead), investigation (lead), methodology (lead), supervision (lead), writing – original draft (equal), writing – review and editing (equal). **Maurizio Pinna:** conceptualization (lead), data curation (lead), formal analysis (lead), funding acquisition (lead), investigation (lead), methodology (lead), supervision (lead), writing – original draft (lead), writing – review and editing (lead).

## Funding

This research was supported by the project titled “Shared BLUE knowledge and skills to sustain BIODIVERSITY in mariculture ‐ BlueDiversity”, code: ITHR0200404, funded by Interreg Italy‐Croatia 2021–2027 Programme First Call.

## Conflicts of Interest

The authors declare no conflicts of interest.

## Supporting information


**Figure S1:** Maximum Likelihood phylogenetic tree built on 96 ASVs of *Callinectes sapidus* together with 27 reference COI sequences representing the phylogenetic diversity of the genus *Callinectes*, and one COI sequence representing *Scylla serrata* as the outgroup.

**Table S1:** List of aquatic species used for testing the forward primer.

## Data Availability

Raw NGS data are available in the NCBI Sequence Read Archive (SRA) under BioProject accession number: PRJNA1459683. https://dataview.ncbi.nlm.nih.gov/object/PRJNA1459683?reviewer=5jiujb1q3o2550t772eqsuugj7. Sanger sequences have been deposited in GenBank under accession number PZ346324.
