# Supplementary material for: Targeted eDNA Survey Enables Consistent Detection of the Invasive Blue Crab in Trans‐Border Adriatic Coastal‐Marine Ecosystems
Source: Ecol Evol. 2026 Oct 12;16(10):e74462. doi: 10.1002/ece3.74462 (PMC13658837; doi:10.1002/ece3.74462)
Supplement: Supplementary file 1 [file ECE3-16-e74462-s001.docx]

**Figure S1.** Maximum Likelihood phylogenetic tree built on 96 ASVs of *Callinectes sapidus* together with 27 reference COI sequences representing the phylogenetic diversity of the genus *Callinectes*, and one COI sequence representing *Scylla serrata* as the outgroup.
