# Supplementary material for: Targeted eDNA Survey Enables Consistent Detection of the Invasive Blue Crab in Trans‐Border Adriatic Coastal‐Marine Ecosystems
Source: Ecol Evol. 2026 Oct 12;16(10):e74462. doi: 10.1002/ece3.74462 (PMC13658837; doi:10.1002/ece3.74462)
Supplement: Supplementary file 2 [file ECE3-16-e74462-s002.docx]

**Table S1.** List of aquatic species used for testing the forward primer.

| **Kingdom** | **Phylum** | **Class** | **Order** | **Family** | **Genus** | **Species** | **Scientific Name** |
| --- | --- | --- | --- | --- | --- | --- | --- |
| Animalia | Mollusca | Bivalvia | Cardiida | Semelidae | *Abra* | *Abra alba* | *Abra alba* (W. Wood, 1802) |
| Animalia | Mollusca | Bivalvia | Cardiida | Semelidae | *Abra* | *Abra nitida* | *Abra nitida* (O. F. Müller, 1776) |
| Animalia | Mollusca | Bivalvia | Cardiida | Semelidae | *Abra* | *Abra prismatica* | *Abra prismatica* (Montagu, 1808) |
| Animalia | Mollusca | Bivalvia | Cardiida | Cardiidae | *Acanthocardia* | *Acanthocardia aculeata* | *Acanthocardia aculeata* (Linnaeus, 1758) |
| Animalia | Mollusca | Bivalvia | Cardiida | Cardiidae | *Acanthocardia* | *Acanthocardia echinata* | *Acanthocardia echinata* (Linnaeus, 1758) |
| Animalia | Mollusca | Bivalvia | Cardiida | Cardiidae | *Acanthocardia* | *Acanthocardia paucicostata* | *Acanthocardia paucicostata* (G. B. Sowerby II, 1834) |
| Animalia | Mollusca | Polyplacophora | Chitonida | Acanthochitonidae | *Acanthochitona* | *Acanthochitona fascicularis* | *Acanthochitona fascicularis* (Linnaeus, 1767) |
| Animalia | Arthropoda | Malacostraca | Decapoda | Portunidae | *Achelous* | *Achelous hastatus* | *Achelous hastatus* (Linnaeus, 1767) |
| Animalia | Echinodermata | Ophiuroidea | Amphilepidida | Amphiuridae | *Acrocnida* | *Acrocnida brachiata* | *Acrocnida brachiata* (Montagu, 1804) |
| Animalia | Mollusca | Gastropoda |  | Acroloxidae | *Acroloxus* | *Acroloxus lacustris* | *Acroloxus lacustris* (Linnaeus, 1758) |
| Animalia | Annelida | Polychaeta | Sabellida | Sabellidae | *Acromegalomma* | *Acromegalomma vesiculosum* | *Acromegalomma vesiculosum* (Montagu, 1813) |
| Animalia | Mollusca | Gastropoda |  | Acteonidae | *Acteon* | *Acteon tornatilis* | *Acteon tornatilis* (Linnaeus, 1758) |
| Animalia | Cnidaria | Anthozoa | Actiniaria | Hormathiidae | *Calliactis* | *Calliactis palliata* | *Adamsia palliata* (Bohadsch, 1761) |
| Animalia | Mollusca | Bivalvia | Pectinida | Pectinidae | *Aequipecten* | *Aequipecten opercularis* | *Aequipecten opercularis* (Linnaeus, 1758) |
| Animalia | Mollusca | Gastropoda | Aplysiida | Akeridae | *Akera* | *Akera bullata* | *Akera bullata* O. F. Müller, 1776 |
| Animalia | Annelida | Polychaeta | Phyllodocida | Nereididae | *Alitta* | *Alitta succinea* | *Alitta succinea* (Leuckart, 1847) |
| Animalia | Arthropoda | Malacostraca | Decapoda | Alpheidae | *Alpheus* | *Alpheus glaber* | *Alpheus glaber* (Olivi, 1792) |
| Animalia | Bryozoa | Gymnolaemata | Ctenostomatida | Vesiculariidae | *Amathia* | *Amathia gracilis* | *Amathia gracilis* (Leidy, 1855) |
| Animalia | Bryozoa | Gymnolaemata | Ctenostomatida | Vesiculariidae | *Amathia* | *Amathia imbricata* | *Amathia imbricata* (Adams, 1800) |
| Animalia | Bryozoa | Gymnolaemata | Ctenostomatida | Vesiculariidae | *Amathia* | *Amathia semiconvoluta* | *Amathia semiconvoluta* Lamouroux, 1824 |
| Animalia | Arthropoda | Malacostraca | Amphipoda | Ampeliscidae | *Ampelisca* | *Ampelisca brevicornis* | *Ampelisca brevicornis* (A. Costa, 1853) |
| Animalia | Arthropoda | Malacostraca | Amphipoda | Ampeliscidae | *Ampelisca* | *Ampelisca diadema* | *Ampelisca diadema* (A. Costa, 1853) |
| Animalia | Arthropoda | Malacostraca | Amphipoda | Ampeliscidae | *Ampelisca* | *Ampelisca sarsi* | *Ampelisca sarsi* Chevreux, 1888 |
| Animalia | Arthropoda | Malacostraca | Amphipoda | Ampeliscidae | *Ampelisca* | *Ampelisca spinipes* | *Ampelisca spinipes* Boeck, 1861 |
| Animalia | Arthropoda | Malacostraca | Amphipoda | Ampeliscidae | *Ampelisca* | *Ampelisca typica* | *Ampelisca typica* (Spence Bate, 1857) |
| Animalia | Annelida | Polychaeta |  | Ampharetidae | *Ampharete* | *Ampharete acutifrons* | *Ampharete acutifrons* (Grube, 1860) |
| Animalia | Annelida | Polychaeta |  | Ampharetidae | *Ampharete* | *Ampharete octocirrata* | *Ampharete octocirrata* (Sars, 1835) |
| Animalia | Arthropoda | Thecostraca | Balanomorpha | Balanidae | *Amphibalanus* | *Amphibalanus amphitrite* | *Amphibalanus amphitrite* (Darwin, 1854) |
| Animalia | Arthropoda | Thecostraca | Balanomorpha | Balanidae | *Amphibalanus* | *Amphibalanus improvisus* | *Amphibalanus improvisus* (Darwin, 1854) |
| Animalia | Annelida | Polychaeta |  | Pectinariidae | *Amphictene* | *Amphictene auricoma* | *Amphictene auricoma* (O.F. Müller, 1776) |
| Animalia | Echinodermata | Ophiuroidea | Amphilepidida | Amphiuridae | *Amphiura* | *Amphiura chiajei* | *Amphiura chiajei* Forbes, 1843 |
| Animalia | Echinodermata | Ophiuroidea | Amphilepidida | Amphiuridae | *Amphiura* | *Amphiura filiformis* | *Amphiura filiformis* (O.F. Müller, 1776) |
| Animalia | Arthropoda | Malacostraca | Decapoda | Paguridae | *Anapagurus* | *Anapagurus bicorniger* | *Anapagurus bicorniger* A. Milne-Edwards & Bouvier, 1892 |
| Animalia | Arthropoda | Malacostraca | Decapoda | Paguridae | *Anapagurus* | *Anapagurus breviaculeatus* | *Anapagurus breviaculeatus* Fenizia, 1937 |
| Animalia | Arthropoda | Malacostraca | Decapoda | Paguridae | *Anapagurus* | *Anapagurus chiroacanthus* | *Anapagurus chiroacanthus* (Lilljeborg, 1856) |
| Animalia | Annelida | Polychaeta | Phyllodocida | Pilargidae | *Ancistrosyllis* | *Ancistrosyllis groenlandica* | *Ancistrosyllis groenlandica* McIntosh, 1878 |
| Animalia | Mollusca | Bivalvia | Pectinida | Anomiidae | *Anomia* | *Anomia ephippium* | *Anomia ephippium* Linnaeus, 1758 |
| Animalia | Mollusca | Scaphopoda | Dentaliida | Dentaliidae | *Antalis* | *Antalis agilis* | *Antalis agilis* (M. Sars, 1872) |
| Animalia | Mollusca | Scaphopoda | Dentaliida | Dentaliidae | *Antalis* | *Antalis dentalis* | *Antalis dentalis* (Linnaeus, 1758) |
| Animalia | Mollusca | Scaphopoda | Dentaliida | Dentaliidae | *Antalis* | *Antalis inaequicostata* | *Antalis inaequicostata* (Dautzenberg, 1891) |
| Animalia | Mollusca | Scaphopoda | Dentaliida | Dentaliidae | *Antalis* | *Antalis vulgaris* | *Antalis vulgaris* (da Costa, 1778) |
| Animalia | Annelida | Polychaeta |  | Spionidae | *Aonides* | *Aonides oxycephala* | *Aonides oxycephala* (Sars, 1862) |
| Animalia | Annelida | Polychaeta |  | Spionidae | *Aonides* | *Aonides paucibranchiata* | *Aonides paucibranchiata* Southern, 1914 |
| Animalia | Arthropoda | Malacostraca | Amphipoda | Aoridae | *Aora* | *Aora gracilis* | *Aora gracilis* (Spence Bate, 1857) |
| Animalia | Annelida | Polychaeta |  | Cirratulidae | *Aphelochaeta* | *Aphelochaeta filiformis* | *Aphelochaeta filiformis* (Keferstein, 1862) |
| Animalia | Annelida | Polychaeta | Phyllodocida | Aphroditidae | *Aphrodita* | *Aphrodita aculeata* | *Aphrodita aculeata* Linnaeus, 1758 |
| Animalia | Mollusca | Gastropoda | Neogastropoda | Pisaniidae | *Aplus* | *Aplus dorbignyi* | *Aplus dorbignyi* (Payraudeau, 1826) |
| Animalia | Mollusca | Gastropoda | Neogastropoda | Pisaniidae | *Aplus* | *Aplus scaber* | *Aplus scaber* (Millet, 1865) |
| Animalia | Mollusca | Gastropoda | Littorinimorpha | Aporrhaidae | *Aporrhais* | *Aporrhais pespelecani* | *Aporrhais pespelecani* (Linnaeus, 1758) |
| Animalia | Mollusca | Gastropoda | Neogastropoda | Fasciolariidae | *Aptyxis* | *Aptyxis syracusana* | *Aptyxis syracusana* (Linnaeus, 1758) |
| Animalia | Echinodermata | Echinoidea | Arbacioida | Arbaciidae | *Arbacia* | *Arbacia lixula* | *Arbacia lixula* (Linnaeus, 1758) |
| Animalia | Mollusca | Bivalvia | Arcida | Arcidae | *Arca* | *Arca noae* | *Arca noae* Linnaeus, 1758 |
| Animalia | Mollusca | Bivalvia | Mytilida | Mytilidae | *Arcuatula* | *Arcuatula senhousia* | *Arcuatula senhousia* (W. H. Benson, 1842) |
| Animalia | Annelida | Polychaeta |  | Paraonidae | *Aricidea* | *Aricidea assimilis* | *Aricidea (Acmira)* assimilis Tebble, 1959 |
| Animalia | Annelida | Polychaeta |  | Paraonidae | *Aricidea* | *Aricidea capensis* | *Aricidea (Aricidea)* capensis Day, 1961 |
| Animalia | Annelida | Polychaeta |  | Paraonidae | *Aricidea* | *Aricidea catherinae* | *Aricidea (Acmira)* catherinae Laubier, 1967 |
| Animalia | Annelida | Polychaeta |  | Paraonidae | *Aricidea* | *Aricidea cerrutii* | *Aricidea (Acmira)* cerrutii Laubier, 1966 |
| Animalia | Annelida | Polychaeta |  | Paraonidae | *Aricidea* | *Aricidea claudiae* | *Aricidea (Strelzovia)* claudiae Laubier, 1967 |
| Animalia | Annelida | Polychaeta |  | Paraonidae | *Aricidea* | *Aricidea mariannae* | *Aricidea (Strelzovia)* mariannae Katzmann & Laubier, 1975 |
| Animalia | Annelida | Polychaeta |  | Paraonidae | *Aricidea* | *Aricidea wassi* | *Aricidea (Aricidea)* wassi Pettibone, 1965 |
| Animalia | Mollusca | Bivalvia | Cardiida | Tellinidae | *Asbjornsenia* | *Asbjornsenia pygmaea* | *Asbjornsenia pygmaea* (Lovén, 1846) |
| Animalia | Chordata | Ascidiacea | Phlebobranchia | Ascidiidae | *Ascidia* | *Ascidia conchilega* | *Ascidia conchilega* Müller, 1776 |
| Animalia | Chordata | Ascidiacea | Phlebobranchia | Ascidiidae | *Ascidiella* | *Ascidiella aspersa* | *Ascidiella aspersa* (Müller, 1776) |
| Animalia | Chordata | Ascidiacea | Phlebobranchia | Ascidiidae | *Ascidiella* | *Ascidiella scabra* | *Ascidiella scabra* (Müller, 1776) |
| Animalia | Arthropoda | Malacostraca | Isopoda | Asellidae | *Asellus* | *Asellus aquaticus* | *Asellus aquaticus* (Linnaeus, 1758) |
| Animalia | Echinodermata | Asteroidea | Paxillosida | Astropectinidae | *Astropecten* | *Astropecten irregularis* | *Astropecten irregularis* (Pennant, 1777) |
| Animalia | Echinodermata | Asteroidea | Paxillosida | Astropectinidae | *Astropecten* | *Astropecten jonstoni* | *Astropecten jonstoni* (Delle Chiaje, 1827) |
| Animalia | Echinodermata | Asteroidea | Paxillosida | Astropectinidae | *Astropecten* | *Astropecten spinulosus* | *Astropecten spinulosus* (Philippi, 1837) |
| Animalia | Arthropoda | Malacostraca | Decapoda | Alpheidae | *Athanas* | *Athanas nitescens* | *Athanas nitescens* (Leach, 1813) |
| Animalia | Arthropoda | Malacostraca | Decapoda | Atyidae | *Atyaephyra* | *Atyaephyra desmarestii* | *Atyaephyra desmarestii* (Millet, 1831) |
| Animalia | Cnidaria | Anthozoa | Actiniaria | Actiniidae | *Bunodactis* | *Bunodactis verrucosa* | *Aulactinia verrucosa* (Pennant, 1777) |
| Animalia | Arthropoda | Thecostraca | Balanomorpha | Balanidae | *Balanus* | *Balanus trigonus* | *Balanus trigonus* Darwin, 1854 |
| Animalia | Mollusca | Bivalvia | Arcida | Arcidae | *Barbatia* | *Barbatia barbata* | *Barbatia barbata* (Linnaeus, 1758) |
| Animalia | Entoprocta |  | Solitaria | Barentsiidae | *Barentsia* | *Barentsia gracilis* | *Barentsia gracilis* (M. Sars, 1835) |
| Animalia | Arthropoda | Malacostraca | Decapoda | Polybiidae | *Bathynectes* | *Bathynectes maravigna* | *Bathynectes maravigna* (Prestandrea, 1839) |
| Animalia | Arthropoda | Malacostraca | Amphipoda | Bathyporeiidae | *Bathyporeia* | *Bathyporeia elegans* | *Bathyporeia elegans* Watkin, 1938 |
| Animalia | Arthropoda | Malacostraca | Amphipoda | Bathyporeiidae | *Bathyporeia* | *Bathyporeia guilliamsoniana* | *Bathyporeia guilliamsoniana* (Spence Bate, 1857) |
| Animalia | Bryozoa | Gymnolaemata | Cheilostomatida | Beaniidae | *Beania* | *Beania magellanica* | *Beania magellanica* (Busk, 1852) |
| Animalia | Mollusca | Gastropoda | Littorinimorpha | Bithyniidae | *Bithynia* | *Bithynia tentaculata* | *Bithynia tentaculata* (Linnaeus, 1758) |
| Animalia | Mollusca | Gastropoda |  | Cerithiidae | *Bittium* | *Bittium latreillii* | *Bittium latreillii* (Payraudeau, 1826) |
| Animalia | Mollusca | Gastropoda |  | Cerithiidae | *Bittium* | *Bittium reticulatum* | *Bittium reticulatum* (da Costa, 1778) |
| Animalia | Arthropoda | Malacostraca | Cumacea | Bodotriidae | *Bodotria* | *Bodotria scorpioides* | *Bodotria scorpioides* (Montagu, 1804) |
| Animalia | Mollusca | Gastropoda | Neogastropoda | Muricidae | *Bolinus* | *Bolinus brandaris* | *Bolinus brandaris* (Linnaeus, 1758) |
| Animalia | Mollusca | Gastropoda | Trochida | Turbinidae | *Bolma* | *Bolma rugosa* | *Bolma rugosa* (Linnaeus, 1767) |
| Animalia | Mollusca | Bivalvia | Venerida | Lasaeidae | *Bornia* | *Bornia sebetia* | *Bornia sebetia* (O. G. Costa, 1830) |
| Animalia | Chordata | Ascidiacea | Stolidobranchia | Styelidae | *Botryllus* | *Botryllus schlosseri* | *Botryllus schlosseri* (Pallas, 1766) |
| Animalia | Mollusca | Bivalvia | Mytilida | Mytilidae | *Brachidontes* | *Brachidontes pharaonis* | *Brachidontes pharaonis* (P. Fischer, 1870) |
| Animalia | Annelida | Polychaeta |  | Flabelligeridae | *Bradabyssa* | *Bradabyssa villosa* | *Bradabyssa villosa* (Rathke, 1843) |
| Animalia | Chordata | Leptocardii |  | Branchiostomatidae | *Branchiostoma* | *Branchiostoma lanceolatum* | *Branchiostoma lanceolatum* (Pallas, 1774) |
| Animalia | Annelida | Polychaeta | Phyllodocida | Syllidae | *Branchiosyllis* | *Branchiosyllis cirropunctata* | *Branchiosyllis cirropunctata* (Michel, 1909) |
| Animalia | Bryozoa | Gymnolaemata | Cheilostomatida | Bugulidae | *Bugula* | *Bugula neritina* | *Bugula neritina* (Linnaeus, 1758) |
| Animalia | Bryozoa | Gymnolaemata | Cheilostomatida | Bugulidae | *Bugulina* | *Bugulina fulva* | *Bugulina fulva* (Ryland, 1960) |
| Animalia | Bryozoa | Gymnolaemata | Cheilostomatida | Bugulidae | *Bugulina* | *Bugulina simplex* | *Bugulina simplex* (Hincks, 1886) |
| Animalia | Bryozoa | Gymnolaemata | Cheilostomatida | Bugulidae | *Bugulina* | *Bugulina stolonifera* | *Bugulina stolonifera* (Ryland, 1960) |
| Animalia | Bryozoa | Gymnolaemata | Ctenostomatida | Victorellidae | *Bulbella* | *Bulbella abscondita* | *Bulbella abscondita* Braem, 1951 |
| Animalia | Mollusca | Gastropoda | Littorinimorpha | Caecidae | *Caecum* | *Caecum trachea* | *Caecum trachea* (Montagu, 1803) |
| Animalia | Arthropoda | Malacostraca | Decapoda | Portunidae | *Callinectes* | *Callinectes danae* | *Callinectes danae* Smith, 1869 |
| Animalia | Arthropoda | Malacostraca | Decapoda | Portunidae | *Callinectes* | *Callinectes sapidus* | *Callinectes sapidus* Rathbun, 1896 |
| Animalia | Mollusca | Gastropoda | Trochida | Calliostomatidae | *Calliostoma* | *Calliostoma laugieri* | *Calliostoma laugieri* (Payraudeau, 1826) |
| Animalia | Mollusca | Gastropoda | Trochida | Calliostomatidae | *Calliostoma* | *Calliostoma laugieri* | *Calliostoma virescens* Coen, 1933 |
| Animalia | Mollusca | Bivalvia | Venerida | Veneridae | *Callista* | *Callista chione* | *Callista chione* (Linnaeus, 1758) |
| Animalia | Mollusca | Gastropoda | Nudibranchia | Calmidae | *Calma* | *Calma glaucoides* | *Calma glaucoides* (Alder & Hancock, 1854) |
| Animalia | Mollusca | Gastropoda | Littorinimorpha | Calyptraeidae | *Calyptraea* | *Calyptraea chinensis* | *Calyptraea chinensis* (Linnaeus, 1758) |
| Animalia | Annelida | Polychaeta |  | Capitellidae | *Capitella* | *Capitella capitata* | *Capitella capitata* (Fabricius, 1780) |
| Animalia | Arthropoda | Malacostraca | Amphipoda | Caprellidae | *Caprella* | *Caprella acanthifera* | *Caprella acanthifera* Leach, 1814 |
| Animalia | Arthropoda | Malacostraca | Amphipoda | Caprellidae | *Caprella* | *Caprella equilibra* | *Caprella equilibra* Say, 1818 |
| Animalia | Arthropoda | Malacostraca | Amphipoda | Caprellidae | *Caprella* | *Caprella scaura* | *Caprella scaura* Templeton, 1836 |
| Animalia | Arthropoda | Malacostraca | Decapoda | Carcinidae | *Carcinus* | *Carcinus aestuarii* | *Carcinus aestuarii* Nardo, 1847 |
| Animalia | Arthropoda | Malacostraca | Decapoda | Carcinidae | *Carcinus* | *Carcinus maenas* | *Carcinus maenas* (Linnaeus, 1758) |
| Animalia | Mollusca | Bivalvia |  |  | *Cardita* | *Cardita calyculata* | *Cardita calyculata* (Linnaeus, 1758) |
| Animalia | Mollusca | Bivalvia |  | Carditidae | *Cardites* | *Cardites antiquatus* | *Cardites antiquatus* (Linnaeus, 1758) |
| Animalia | Mollusca | Bivalvia | Cardiida | Cardiidae | *Cerastoderma* | *Cerastoderma edule* | *Cerastoderma edule* (Linnaeus, 1758) |
| Animalia | Mollusca | Bivalvia | Cardiida | Cardiidae | *Cerastoderma* | *Cerastoderma glaucum* | *Cerastoderma glaucum* (Bruguière, 1789) |
| Animalia | Cnidaria | Anthozoa | Actiniaria | Sagartiidae | *Cereus* | *Cereus pedunculatus* | *Cereus pedunculatus* (Pennant, 1777) |
| Animalia | Cnidaria | Anthozoa | Ceriantharia | Cerianthidae | *Cerianthus* | *Cerianthus membranaceus* | *Cerianthus membranaceus* (Gmelin, 1791) |
| Animalia | Mollusca | Gastropoda |  | Potamididae | *Pirenella* |  | *Cerithideopsilla conica* (Blainville, 1829) |
| Animalia | Arthropoda | Malacostraca | Decapoda | Paguridae | *Cestopagurus* | *Cestopagurus timidus* | *Cestopagurus timidus* (Roux, 1830) |
| Animalia | Annelida | Polychaeta |  | Chaetopteridae | *Chaetopterus* | *Chaetopterus variopedatus* | *Chaetopterus variopedatus* (Renier, 1804) |
| Animalia | Annelida | Polychaeta |  | Cirratulidae | *Chaetozone* | *Chaetozone setosa* | *Chaetozone setosa* Malmgren, 1867 |
| Animalia | Mollusca | Bivalvia | Venerida | Chamidae | *Chama* | *Chama gryphoides* | *Chama gryphoides* Linnaeus, 1758 |
| Animalia | Bryozoa | Gymnolaemata | Cheilostomatida | Chorizoporidae | *Chorizopora* | *Chorizopora brongniartii* | *Chorizopora brongniartii* (Audouin, 1826) |
| Animalia | Arthropoda | Thecostraca | Balanomorpha | Chthamalidae | *Chthamalus* | *Chthamalus stellatus* | *Chthamalus stellatus* (Poli, 1791) |
| Animalia | Annelida | Polychaeta |  | Cirratulidae | *Cirratulus* | *Cirratulus cirratus* | *Cirratulus cirratus* (O. F. Müller, 1776) |
| Animalia | Annelida | Polychaeta |  | Cirratulidae | *Cirriformia* | *Cirriformia tentaculata* | *Cirriformia tentaculata* (Montagu, 1808) |
| Animalia | Cnidaria | Anthozoa | Scleractinia | Cladocoridae | *Cladocora* | *Cladocora caespitosa* | *Cladocora caespitosa* (Linnaeus, 1767) |
| Animalia | Mollusca | Gastropoda | Trochida | Trochidae | *Clanculus* | *Clanculus jussieui* | *Clanculus jussieui* (Payraudeau, 1826) |
| Animalia | Porifera | Demospongiae | Poecilosclerida | Microcionidae | *Clathria* | *Clathria virgultosa* | *Clathria (Thalysias)* virgultosa (Esper, 1806) |
| Animalia | Chordata | Ascidiacea | Aplousobranchia | Clavelinidae | *Clavelina* | *Clavelina oblonga* | *Clavelina oblonga* Herdman, 1880 |
| Animalia | Arthropoda | Malacostraca | Decapoda | Diogenidae | *Clibanarius* | *Clibanarius erythropus* | *Clibanarius erythropus* (Latreille, 1818) |
| Animalia | Porifera | Demospongiae | Clionaida | Clionaidae | *Cliona* | *Cliona viridis* | *Cliona viridis* (Schmidt, 1862) |
| Animalia | Cnidaria | Hydrozoa | Leptothecata | Campanulariidae | *Clytia* | *Clytia linearis* | *Clytia linearis* (Thorneley, 1900) |
| Animalia | Mollusca | Gastropoda | Neogastropoda | Columbellidae | *Columbella* | *Columbella rustica* | *Columbella rustica* (Linnaeus, 1758) |
| Animalia | Mollusca | Gastropoda | Neogastropoda | Clathurellidae | *Pleurotomoides* | *Pleurotomoides gracilis* | *Comarmondia gracilis* (Montagu, 1803) |
| Animalia | Mollusca | Gastropoda | Neogastropoda | Conidae | *Conus* | *Conus ventricosus* | *Conus ventricosus* Gmelin, 1791 |
| Animalia | Cnidaria | Hydrozoa | Anthoathecata | Cordylophoridae | *Cordylophora* | *Cordylophora caspia* | *Cordylophora caspia* (Pallas, 1771) |
| Animalia | Arthropoda | Malacostraca | Amphipoda | Corophiidae | *Corophium* | *Corophium volutator* | *Corophium volutator* (Pallas, 1766) |
| Animalia | Arthropoda | Malacostraca | Decapoda | Corystidae | *Corystes* | *Corystes cassivelaunus* | *Corystes cassivelaunus* (Pennant, 1777) |
| Animalia | Arthropoda | Malacostraca | Decapoda | Crangonidae | *Crangon* | *Crangon crangon* | *Crangon crangon* (Linnaeus, 1758) |
| Animalia | Arthropoda | Malacostraca | Amphipoda | Corophiidae | *Crassicorophium* | *Crassicorophium bonellii* | *Crassicorophium bonellii* (H. Milne Edwards, 1830) |
| Animalia | Bryozoa | Gymnolaemata | Cheilostomatida | Cryptosulidae | *Cryptosula* | *Cryptosula pallasiana* | *Cryptosula pallasiana* (Moll, 1803) |
| Animalia | Mollusca | Gastropoda |  | Cylichnidae | *Cylichna* | *Cylichna cylindracea* | *Cylichna cylindracea* (Pennant, 1777) |
| Animalia | Arthropoda | Malacostraca | Decapoda | Diogenidae | *Dardanus* | *Dardanus arrosor* | *Dardanus arrosor* (J. F. W. Herbst, 1796) |
| Animalia | Arthropoda | Malacostraca | Decapoda | Diogenidae | *Dardanus* | *Dardanus calidus* | *Dardanus calidus* (Risso, 1827) |
| Animalia | Mollusca | Gastropoda |  | Dendrodorididae | *Dendrodoris* | *Dendrodoris grandiflora* | *Dendrodoris grandiflora* (Rapp, 1827) |
| Animalia | Arthropoda | Malacostraca | Decapoda | Parthenopidae | *Derilambrus* | *Derilambrus angulifrons* | *Derilambrus angulifrons* (Latreille, 1825) |
| Animalia | Arthropoda | Malacostraca | Amphipoda | Dexaminidae | *Dexamine* | *Dexamine spiniventris* | *Dexamine spiniventris* (A. Costa, 1853) |
| Animalia | Arthropoda | Malacostraca | Amphipoda | Dexaminidae | *Dexamine* | *Dexamine spinosa* | *Dexamine spinosa* (Montagu, 1813) |
| Animalia | Cnidaria | Anthozoa | Actiniaria | Diadumenidae | *Diadumene* | *Diadumene lineata* | *Diadumene lineata* (Verrill, 1869) |
| Animalia | Annelida | Polychaeta | Sabellida | Sabellidae | *Dialychone* | *Dialychone collaris* | *Dialychone collaris* (Langerhans, 1880) |
| Animalia | Arthropoda | Malacostraca | Cumacea | Diastylidae | *Diastylis* | *Diastylis rugosa* | *Diastylis rugosa* Sars, 1865 |
| Animalia | Chordata | Ascidiacea | Aplousobranchia | Didemnidae | *Didemnum* | *Didemnum candidum* | *Didemnum candidum* Savigny, 1816 |
| Animalia | Mollusca | Gastropoda | Lepetellida | Fissurellidae | *Diodora* | *Diodora graeca* | *Diodora graeca* (Linnaeus, 1758) |
| Animalia | Arthropoda | Malacostraca | Decapoda | Diogenidae | *Diogenes* | *Diogenes pugilator* | *Diogenes pugilator* (Roux, 1828) |
| Animalia | Annelida | Polychaeta |  | Flabelligeridae | *Diplocirrus* | *Diplocirrus glaucus* | *Diplocirrus glaucus* (Malmgren, 1867) |
| Animalia | Bryozoa | Stenolaemata | Cyclostomatida | Lichenoporidae | *Disporella* | *Disporella hispida* | *Disporella hispida* (Fleming, 1828) |
| Animalia | Annelida | Polychaeta | Sabellida | Serpulidae | *Ditrupa* | *Ditrupa arietina* | *Ditrupa arietina* (O. F. Müller, 1776) |
| Animalia | Annelida | Polychaeta |  | Cirratulidae | *Dodecaceria* | *Dodecaceria concharum* | *Dodecaceria concharum* Örsted, 1843 |
| Animalia | Mollusca | Bivalvia | Venerida | Mesodesmatidae | *Donacilla* | *Donacilla cornea* | *Donacilla cornea* (Poli, 1791) |
| Animalia | Mollusca | Bivalvia | Cardiida | Donacidae | *Donax* | *Donax trunculus* | *Donax trunculus* Linnaeus, 1758 |
| Animalia | Mollusca | Bivalvia | Cardiida | Donacidae | *Donax* | *Donax variegatus* | *Donax variegatus* (Gmelin, 1791) |
| Animalia | Mollusca | Gastropoda |  | Dendrodorididae | *Doriopsilla* | *Doriopsilla areolata* | *Doriopsilla areolata* Bergh, 1880 |
| Animalia | Mollusca | Bivalvia | Venerida | Veneridae | *Dosinia* | *Dosinia exoleta* | *Dosinia exoleta* (Linnaeus, 1758) |
| Animalia | Mollusca | Bivalvia | Venerida | Veneridae | *Dosinia* | *Dosinia lupinus* | *Dosinia lupinus* (Linnaeus, 1758) |
| Animalia | Annelida | Polychaeta | Eunicida | Oenonidae | *Drilonereis* | *Drilonereis filum* | *Drilonereis filum* (Claparède, 1868) |
| Animalia | Arthropoda | Malacostraca | Isopoda | Sphaeromatidae | *Dynamene* | *Dynamene bidentata* | *Dynamene bidentata* (Adams, 1800) |
| Animalia | Arthropoda | Malacostraca | Decapoda | Panopeidae | *Dyspanopeus* | *Dyspanopeus sayi* | *Dyspanopeus sayi* (Smith, 1869) |
| Animalia | Arthropoda | Malacostraca | Decapoda | Leucosiidae | *Ebalia* | *Ebalia cranchii* | *Ebalia cranchii* Leach, 1817 |
| Animalia | Arthropoda | Malacostraca | Decapoda | Leucosiidae | *Ebalia* | *Ebalia edwardsii* | *Ebalia edwardsii* O.G. Costa, 1838 |
| Animalia | Arthropoda | Malacostraca | Decapoda | Leucosiidae | *Ebalia* | *Ebalia tuberosa* | *Ebalia tuberosa* (Pennant, 1777) |
| Animalia | Arthropoda | Malacostraca | Decapoda | Leucosiidae | *Ebalia* | *Ebalia tumefacta* | *Ebalia tumefacta* (Montagu, 1808) |
| Animalia | Mollusca | Gastropoda | Neogastropoda | Costellariidae | *Ebenomitra* | *Ebenomitra ebenus* | *Ebenomitra ebenus* (Lamarck, 1811) |
| Animalia | Mollusca | Gastropoda | Neogastropoda | Costellariidae | *Ebenomitra* | *Ebenomitra tricolor* | *Ebenomitra tricolor* (Gmelin, 1791) |
| Animalia | Echinodermata | Echinoidea |  | Fibulariidae | *Echinocyamus* | *Echinocyamus pusillus* | *Echinocyamus pusillus* (O.F. Müller, 1776) |
| Animalia | Arthropoda | Malacostraca | Amphipoda | Gammaridae | *Pectenogammarus* | *Pectenogammarus stocki* | *Echinogammarus stocki* G. Karaman, 1970 |
| Animalia | Cnidaria | Hydrozoa | Anthoathecata | Tubulariidae | *Ectopleura* | *Ectopleura crocea* | *Ectopleura crocea* (Agassiz, 1862) |
| Animalia | Arthropoda | Malacostraca | Amphipoda | Maeridae | *Elasmopus* | *Elasmopus rapax* | *Elasmopus rapax* A. Costa, 1853 |
| Animalia | Mollusca | Gastropoda |  | Plakobranchidae | *Elysia* | *Elysia timida* | *Elysia timida* (Risso, 1818) |
| Animalia | Mollusca | Bivalvia | Adapedonta | Pharidae | *Ensis* | *Ensis ensis* | *Ensis ensis* (Linnaeus, 1758) |
| Animalia | Mollusca | Bivalvia | Adapedonta | Pharidae | *Ensis* | *Ensis siliqua* | *Ensis siliqua* (Linnaeus, 1758) |
| Animalia | Mollusca | Gastropoda |  | Epitoniidae | *Epitonium* | *Epitonium clathrus* | *Epitonium clathrus* (Linnaeus, 1758) |
| Animalia | Arthropoda | Malacostraca | Amphipoda | Ischyroceridae | *Ericthonius* | *Ericthonius brasiliensis* | *Ericthonius brasiliensis* (Dana, 1853) |
| Animalia | Arthropoda | Malacostraca | Amphipoda | Ischyroceridae | *Ericthonius* | *Ericthonius difformis* | *Ericthonius difformis* H. Milne Edwards, 1830 |
| Animalia | Arthropoda | Malacostraca | Amphipoda | Ischyroceridae | *Ericthonius* | *Ericthonius punctatus* | *Ericthonius punctatus* (Spence Bate, 1857) |
| Animalia | Arthropoda | Malacostraca | Decapoda | Eriphiidae | *Eriphia* | *Eriphia verrucosa* | *Eriphia verrucosa* (Forskål, 1775) |
| Animalia | Bryozoa | Gymnolaemata | Cheilostomatida | Exochellidae | *Escharoides* | *Escharoides coccinea* | *Escharoides coccinea* (Abildgaard, 1806) |
| Animalia | Annelida | Polychaeta | Phyllodocida | Phyllodocidae | *Eteone* | *Eteone longa* | *Eteone longa* (Fabricius, 1780) |
| Animalia | Arthropoda | Malacostraca | Decapoda | Ethusidae | *Ethusa* | *Ethusa mascarone* | *Ethusa mascarone* (Herbst, 1785) |
| Animalia | Annelida | Polychaeta | Sabellida | Sabellidae | *Euchone* | *Euchone rosea* | *Euchone rosea* Langerhans, 1884 |
| Animalia | Annelida | Polychaeta |  | Maldanidae | *Euclymene* | *Euclymene oerstedii* | *Euclymene oerstedi* [auct. misspelling for oerstedii] |
| Animalia | Cnidaria | Hydrozoa | Anthoathecata | Eudendriidae | *Eudendrium* | *Eudendrium racemosum* | *Eudendrium racemosum* (Cavolini, 1785) |
| Animalia | Annelida | Polychaeta | Phyllodocida | Phyllodocidae | *Eulalia* | *Eulalia viridis* | *Eulalia viridis* (Linnaeus, 1767) |
| Animalia | Annelida | Polychaeta | Phyllodocida | Phyllodocidae | *Eumida* | *Eumida sanguinea* | *Eumida sanguinea* (Örsted, 1843) |
| Animalia | Annelida | Polychaeta | Phyllodocida | Nereididae | *Eunereis* | *Eunereis longissima* | *Eunereis longissima* (Johnston, 1840) |
| Animalia | Annelida | Polychaeta | Eunicida | Eunicidae | *Eunice* | *Eunice vittata* | *Eunice vittata* (Delle Chiaje, 1828) |
| Animalia | Annelida | Polychaeta | Amphinomida | Euphrosinidae | *Euphrosine* | *Euphrosine foliosa* | *Euphrosine foliosa* Audouin & H Milne Edwards, 1833 |
| Animalia | Annelida | Polychaeta |  | Terebellidae | *Eupolymnia* | *Eupolymnia nebulosa* | *Eupolymnia nebulosa* (Montagu, 1819) |
| Animalia | Arthropoda | Malacostraca | Isopoda | Cirolanidae | *Eurydice* | *Eurydice pulchra* | *Eurydice pulchra* Leach, 1816 |
| Animalia | Arthropoda | Malacostraca | Decapoda | Majidae | *Eurynome* | *Eurynome spinosa* | *Eurynome spinosa* Hailstone, 1835 |
| Animalia | Mollusca | Gastropoda | Littorinimorpha | Naticidae | *Euspira* | *Euspira nitida* | *Euspira nitida* (Donovan, 1803) |
| Animalia | Mollusca | Gastropoda | Neogastropoda | Tudiclidae | *Euthria* | *Euthria cornea* | *Euthria cornea* (Linnaeus, 1758) |
| Animalia | Cnidaria | Anthozoa | Actiniaria | Aiptasiidae | *Exaiptasia* | *Exaiptasia diaphana* | *Exaiptasia diaphana* (Rapp, 1829) |
| Animalia | Mollusca | Bivalvia | Cardiida | Tellinidae | *Fabulina* | *Fabulina fabula* | *Fabulina fabula* (Gmelin, 1791) |
| Animalia | Mollusca | Gastropoda |  | Chromodorididae | *Felimare* | *Felimare fontandraui* | *Felimare fontandraui* (Pruvot-Fol, 1951) |
| Animalia | Mollusca | Gastropoda |  | Chromodorididae | *Felimare* | *Felimare picta* | *Felimare picta* (R. A. Philippi, 1836) |
| Animalia | Mollusca | Gastropoda |  | Chromodorididae | *Felimare* | *Felimare villafranca* | *Felimare villafranca* (Risso, 1818) |
| Animalia | Mollusca | Gastropoda | Lepetellida | Fissurellidae | *Fissurella* | *Fissurella rosea* | *Fissurella rosea* (Gmelin, 1791) |
| Animalia | Arthropoda | Malacostraca | Decapoda | Galatheidae | *Galathea* | *Galathea intermedia* | *Galathea intermedia* Lilljeborg, 1851 |
| Animalia | Arthropoda | Malacostraca | Decapoda | Galatheidae | *Galathea* | *Galathea nexa* | *Galathea nexa* Embleton, 1834 |
| Animalia | Annelida | Polychaeta | Sabellida | Oweniidae | *Galathowenia* | *Galathowenia oculata* | *Galathowenia oculata* (Zachs, 1923) |
| Animalia | Mollusca | Gastropoda | Littorinimorpha | Cassidae | *Galeodea* | *Galeodea echinophora* | *Galeodea echinophora* (Linnaeus, 1758) |
| Animalia | Arthropoda | Malacostraca | Amphipoda | Nuuanuidae | *Gammarella* | *Gammarella fucicola* | *Gammarella fucicola* (Leach, 1814) |
| Animalia | Arthropoda | Malacostraca | Amphipoda | Photidae | *Gammaropsis* | *Gammaropsis maculata* | *Gammaropsis maculata* (Johnston, 1828) |
| Animalia | Arthropoda | Malacostraca | Amphipoda | Gammaridae | *Gammarus* | *Gammarus crinicornis* | *Gammarus crinicornis* Stock, 1966 |
| Animalia | Arthropoda | Malacostraca | Amphipoda | Gammaridae | *Gammarus* | *Gammarus insensibilis* | *Gammarus insensibilis* Stock, 1966 |
| Animalia | Mollusca | Bivalvia | Cardiida | Psammobiidae | *Gari* | *Gari depressa* | *Gari depressa* (Pennant, 1777) |
| Animalia | Mollusca | Bivalvia | Cardiida | Psammobiidae | *Gari* | *Gari fervensis* | *Gari fervensis* (Gmelin, 1791) |
| Animalia | Mollusca | Bivalvia | Gastrochaenida | Gastrochaenidae | *Rocellaria* | *Rocellaria dubia* | *Gastrochaena dubia* (Pennant, 1777) |
| Animalia | Arthropoda | Malacostraca | Mysida | Mysidae | *Gastrosaccus* | *Gastrosaccus spinifer* | *Gastrosaccus spinifer* (Goës, 1864) |
| Animalia | Echinodermata | Echinoidea |  | Trigonocidaridae | *Genocidaris* | *Genocidaris maculata* | *Genocidaris maculata* A. Agassiz, 1869 |
| Animalia | Porifera | Demospongiae | Tetractinellida | Geodiidae | *Geodia* | *Geodia cydonium* | *Geodia cydonium* (Linnaeus, 1767) |
| Animalia | Mollusca | Bivalvia | Mytilida | Modiolidae | *Modiolus* | *Modiolus adriaticus* | *Gibbomodiola adriatica* (Lamarck, 1819) |
| Animalia | Mollusca | Gastropoda | Trochida | Trochidae | *Steromphala* | *Steromphala albida* | *Gibbula albida* (Gmelin, 1791) |
| Animalia | Mollusca | Gastropoda | Trochida | Trochidae | *Gibbula* | *Gibbula ardens* | *Gibbula ardens* (Salis Marschlins, 1793) |
| Animalia | Mollusca | Gastropoda | Trochida | Trochidae | *Steromphala* | *Steromphala cineraria* | *Gibbula cineraria* (Linnaeus, 1758) |
| Animalia | Mollusca | Gastropoda | Trochida | Trochidae | *Steromphala* | *Steromphala umbilicaris* | *Gibbula umbilicaris* (Linnaeus, 1758) |
| Animalia | Mollusca | Gastropoda | Trochida | Trochidae | *Steromphala* | *Steromphala varia* | *Gibbula varia* (Linnaeus, 1758) |
| Animalia | Arthropoda | Malacostraca | Decapoda | Callianassidae | *Gilvossius* | *Gilvossius tyrrhenus* | *Gilvossius tyrrhenus* (Petagna, 1792) |
| Animalia | Annelida | Polychaeta | Phyllodocida | Glyceridae | *Glycera* | *Glycera alba* | *Glycera alba* (O.F. Müller, 1776) |
| Animalia | Annelida | Polychaeta | Phyllodocida | Glyceridae | *Glycera* | *Glycera capitata* | *Glycera capitata* Örsted, 1842 |
| Animalia | Annelida | Polychaeta | Phyllodocida | Glyceridae | *Glycera* | *Glycera lapidum* | *Glycera lapidum* Quatrefages, 1866 |
| Animalia | Annelida | Polychaeta | Phyllodocida | Glyceridae | *Glycera* | *Glycera tesselata* | *Glycera tesselata* Grube, 1863 |
| Animalia | Annelida | Polychaeta | Phyllodocida | Glyceridae | *Glycera* | *Glycera unicornis* | *Glycera unicornis* Lamarck, 1818 |
| Animalia | Annelida | Polychaeta | Phyllodocida | Goniadidae | *Glycinde* | *Glycinde nordmanni* | *Glycinde nordmanni* (Malmgren, 1866) |
| Animalia | Mollusca | Bivalvia | Arcida | Glycymerididae | *Glycymeris* | *Glycymeris glycymeris* | *Glycymeris glycymeris* (Linnaeus, 1758) |
| Animalia | Mollusca | Bivalvia | Arcida | Glycymerididae | *Glycymeris* | *Glycymeris nummaria* | *Glycymeris nummaria* (Linnaeus, 1758) |
| Animalia | Mollusca | Bivalvia | Arcida | Glycymerididae | *Glycymeris* | *Glycymeris pilosa* | *Glycymeris pilosa* (Linnaeus, 1767) |
| Animalia | Sipuncula | Sipunculidea | Golfingiiformes | Golfingiidae | *Golfingia* | *Golfingia vulgaris* | *Golfingia (Golfingia)* vulgaris (de Blainville, 1827) |
| Animalia | Arthropoda | Malacostraca | Decapoda | Goneplacidae | *Goneplax* | *Goneplax rhomboides* | *Goneplax rhomboides* (Linnaeus, 1758) |
| Animalia | Annelida | Polychaeta | Phyllodocida | Goniadidae | *Goniada* | *Goniada maculata* | *Goniada maculata* Örsted, 1843 |
| Animalia | Arthropoda | Malacostraca | Stomatopoda | Gonodactylidae | *Gonodactylaceus* | *Gonodactylaceus falcatus* | *Gonodactylaceus falcatus* (Forskål, 1775) |
| Animalia | Cnidaria | Hydrozoa | Leptothecata | Campanulariidae | *Gonothyraea* | *Gonothyraea loveni* | *Gonothyraea loveni* (Allman, 1859) |
| Animalia | Mollusca | Gastropoda | Neogastropoda | Fasciolariidae | *Pseudofusus* | *Pseudofusus rostratus* | *Gracilipurpura rostrata* (Olivi, 1792) |
| Animalia | Annelida | Polychaeta | Phyllodocida | Hesionidae | *Gyptis* | *Gyptis propinqua* | *Gyptis propinqua* Marion & Bobretzky, 1875 |
| Animalia | Mollusca | Gastropoda |  | Haminoeidae | *Haminoea* | *Haminoea hydatis* | *Haminoea hydatis* (Linnaeus, 1758) |
| Animalia | Mollusca | Gastropoda |  | Haminoeidae | *Haminoea* | *Haminoea navicula* | *Haminoea navicula* (da Costa, 1778) |
| Animalia | Annelida | Polychaeta | Phyllodocida | Polynoidae | *Harmothoe* | *Harmothoe extenuata* | *Harmothoe extenuata* (Grube, 1840) |
| Animalia | Annelida | Polychaeta | Phyllodocida | Polynoidae | *Harmothoe* | *Harmothoe spinifera* | *Harmothoe spinifera* (Ehlers, 1864) |
| Animalia | Annelida | Polychaeta | Phyllodocida | Nereididae | *Hediste* | *Hediste diversicolor* | *Hediste diversicolor* (O.F. Müller, 1776) |
| Animalia | Annelida | Polychaeta | Phyllodocida | Phyllodocidae | *Hesionura* | *Hesionura elongata* | *Hesionura elongata* (Southern, 1914) |
| Animalia | Annelida | Polychaeta |  | Capitellidae | *Heteromastus* | *Heteromastus filiformis* | *Heteromastus filiformis* (Claparède, 1864) |
| Animalia | Mollusca | Gastropoda | Neogastropoda | Muricidae | *Hexaplex* | *Hexaplex trunculus* | *Hexaplex trunculus* (Linnaeus, 1758) |
| Animalia | Mollusca | Bivalvia | Adapedonta | Hiatellidae | *Hiatella* | *Hiatella arctica* | *Hiatella arctica* (Linnaeus, 1767) |
| Animalia | Echinodermata | Holothuroidea | Holothuriida | Holothuriidae | *Holothuria* | *Holothuria impatiens* | *Holothuria (Thymiosycia)* impatiens (Forsskål, 1775) |
| Animalia | Echinodermata | Holothuroidea | Holothuriida | Holothuriidae | *Holothuria* | *Holothuria poli* | *Holothuria (Roweothuria)* poli Delle Chiaje, 1824 |
| Animalia | Echinodermata | Holothuroidea | Holothuriida | Holothuriidae | *Holothuria* | *Holothuria tubulosa* | *Holothuria (Holothuria)* tubulosa Gmelin, 1791 |
| Animalia | Mollusca | Gastropoda | Littorinimorpha | Iravadiidae | *Hyala* | *Hyala vitrea* | *Hyala vitrea* (Montagu, 1803) |
| Animalia | Annelida | Polychaeta | Eunicida | Onuphidae | *Hyalinoecia* | *Hyalinoecia tubicola* | *Hyalinoecia tubicola* (O.F. Müller, 1776) |
| Animalia | Annelida | Polychaeta | Sabellida | Serpulidae | *Hydroides* | *Hydroides dianthus* | *Hydroides dianthus* (Verrill, 1873) |
| Animalia | Annelida | Polychaeta | Sabellida | Serpulidae | *Hydroides* | *Hydroides elegans* | *Hydroides elegans* (Haswell, 1883) [nomen protectum] |
| Animalia | Porifera | Demospongiae | Suberitida | Halichondriidae | *Hymeniacidon* | *Hymeniacidon perlevis* | *Hymeniacidon perlevis* (Montagu, 1814) |
| Animalia | Mollusca | Gastropoda |  | Chromodorididae | *Hypselodoris* | *Hypselodoris maculosa* | *Hypselodoris maculosa* (Pease, 1871) |
| Animalia | Arthropoda | Malacostraca | Isopoda | Idoteidae | *Idotea* | *Idotea balthica* | *Idotea baltica* Pallas, 1772 |
| Animalia | Arthropoda | Malacostraca | Isopoda | Idoteidae | *Idotea* | *Idotea chelipes* | *Idotea chelipes* (Pallas, 1766) |
| Animalia | Arthropoda | Malacostraca | Decapoda | Leucosiidae | *Ilia* | *Ilia nucleus* | *Ilia nucleus* (Linnaeus, 1758) |
| Animalia | Arthropoda | Malacostraca | Decapoda | Inachidae | *Inachus* | *Inachus communissimus* | *Inachus communissimus* Rizza, 1839 |
| Animalia | Arthropoda | Malacostraca | Decapoda | Inachidae | *Inachus* | *Inachus thoracicus* | *Inachus thoracicus* Roux, 1830 |
| Animalia | Arthropoda | Malacostraca | Amphipoda | Iphimediidae | *Iphimedia* | *Iphimedia obesa* | *Iphimedia obesa* Rathke, 1843 |
| Animalia | Arthropoda | Malacostraca | Cumacea | Bodotriidae | *Iphinoe* | *Iphinoe trispinosa* | *Iphinoe trispinosa* (Goodsir, 1843) |
| Animalia | Porifera | Demospongiae | Dictyoceratida | Irciniidae | *Ircinia* | *Ircinia variabilis* | *Ircinia variabilis* (Schmidt, 1862) |
| Animalia | Mollusca | Bivalvia | Venerida | Veneridae | *Irus* | *Irus irus* | *Irus irus* (Linnaeus, 1758) |
| Animalia | Mollusca | Polyplacophora | Chitonida | Ischnochitonidae | *Ischnochiton* | *Ischnochiton rissoi* | *Ischnochiton rissoi* (Payraudeau, 1826) |
| Animalia | Arthropoda | Malacostraca | Isopoda | Janiridae | *Janira* | *Janira maculosa* | *Janira maculosa* Leach, 1814 |
| Animalia | Annelida | Polychaeta | Sabellida | Sabellidae | *Jasmineira* | *Jasmineira elegans* | *Jasmineira elegans* Saint-Joseph, 1894 |
| Animalia | Arthropoda | Malacostraca | Amphipoda | Ischyroceridae | *Jassa* | *Jassa marmorata* | *Jassa marmorata* Holmes, 1905 |
| Animalia | Mollusca | Gastropoda | Trochida | Trochidae | *Jujubinus* | *Jujubinus exasperatus* | *Jujubinus exasperatus* (Pennant, 1777) |
| Animalia | Mollusca | Gastropoda | Trochida | Trochidae | *Jujubinus* | *Jujubinus striatus* | *Jujubinus striatus* (Linnaeus, 1758) |
| Animalia | Mollusca | Bivalvia | Venerida | Lasaeidae | *Kellia* | *Kellia suborbicularis* | *Kellia suborbicularis* (Montagu, 1803) |
| Animalia | Cnidaria | Hydrozoa | Leptothecata | Kirchenpaueriidae | *Kirchenpaueria* | *Kirchenpaueria halecioides* | *Kirchenpaueria halecioides* (Alder, 1859) |
| Animalia | Cnidaria | Hydrozoa | Leptothecata | Kirchenpaueriidae | *Kirchenpaueria* | *Kirchenpaueria pinnata* | *Kirchenpaueria pinnata* (Linnaeus, 1758) |
| Animalia | Mollusca | Bivalvia |  |  | *Kurtiella* | *Kurtiella bidentata* | *Kurtiella bidentata* (Montagu, 1803) |
| Animalia | Annelida | Polychaeta | Phyllodocida | Sigalionidae | *Labioleanira* | *Labioleanira yhleni* | *Labioleanira yhleni* (Malmgren, 1867) |
| Animalia | Annelida | Polychaeta | Phyllodocida | Aphroditidae | *Laetmonice* | *Laetmonice hystrix* | *Laetmonice hystrix* (Savigny in Lamarck, 1818) |
| Animalia | Annelida | Polychaeta |  | Pectinariidae | *Lagis* | *Lagis koreni* | *Lagis koreni* Malmgren, 1866 |
| Animalia | Annelida | Polychaeta |  | Terebellidae | *Lanice* | *Lanice conchilega* | *Lanice conchilega* (Pallas, 1766) |
| Animalia | Cnidaria | Hydrozoa | Leptothecata | Campanulariidae | *Laomedea* | *Laomedea angulata* | *Laomedea angulata* Hincks, 1861 |
| Animalia | Annelida | Polychaeta |  | Spionidae | *Laonice* | *Laonice cirrata* | *Laonice cirrata* (M. Sars, 1851) |
| Animalia | Arthropoda | Copepoda |  | Laophontidae | *Laophonte* | *Laophonte cornuta* | *Laophonte cornuta* Philippi, 1840 |
| Animalia | Annelida | Polychaeta |  | Maldanidae | *Leiochone* | *Leiochone leiopygos* | *Leiochone leiopygos* (Grube, 1860) |
| Animalia | Mollusca | Polyplacophora | Chitonida | Tonicellidae | *Lepidochitona* | *Lepidochitona caprearum* | *Lepidochitona caprearum* (Scacchi, 1836) |
| Animalia | Mollusca | Polyplacophora | Chitonida | Tonicellidae | *Lepidochitona* | *Lepidochitona cinerea* | *Lepidochitona cinerea* (Linnaeus, 1767) |
| Animalia | Annelida | Polychaeta | Phyllodocida | Polynoidae | *Lepidonotus* | *Lepidonotus squamatus* | *Lepidonotus squamatus* (Linnaeus, 1758) |
| Animalia | Arthropoda | Insecta | Lepidoptera | Noctuidae | *Leucania* | *Leucania humidicola* | *Leucania solita* Walker, 1856 |
| Animalia | Annelida | Polychaeta |  | Paraonidae | *Levinsenia* | *Levinsenia gracilis* | *Levinsenia gracilis* (Tauber, 1879) |
| Animalia | Mollusca | Gastropoda | Littorinimorpha | Annulariidae | *Diplopoma* | *Diplopoma decussatum* | *Licina decussata* (Lamarck, 1822) |
| Animalia | Arthropoda | Malacostraca | Isopoda | Ligiidae | *Ligia* | *Ligia italica* | *Ligia italica* Fabricius, 1798 |
| Animalia | Mollusca | Bivalvia | Limida | Limidae | *Limaria* | *Limaria loscombi* | *Lima loscombi* G. B. Sowerby I, 1823 |
| Animalia | Mollusca | Bivalvia | Limida | Limidae | *Limaria* | *Limaria tuberculata* | *Limaria tuberculata* (Olivi, 1792) |
| Animalia | Arthropoda | Malacostraca | Decapoda | Polybiidae | *Polybius* | *Polybius bolivari* | *Liocarcinus bolivari* (Zariquiey Álvarez, 1948) |
| Animalia | Arthropoda | Malacostraca | Decapoda | Polybiidae | *Polybius* | *Polybius corrugatus* | *Liocarcinus corrugatus* (Pennant, 1777) |
| Animalia | Arthropoda | Malacostraca | Decapoda | Polybiidae | *Polybius* | *Polybius depurator* | *Liocarcinus depurator* (Linnaeus, 1758) |
| Animalia | Arthropoda | Malacostraca | Decapoda | Polybiidae | *Polybius* | *Polybius maculatus* | *Liocarcinus maculatus* (Risso, 1827) |
| Animalia | Arthropoda | Malacostraca | Decapoda | Polybiidae | *Polybius* | *Polybius navigator* | *Liocarcinus navigator* (Herbst, 1794) |
| Animalia | Arthropoda | Malacostraca | Decapoda | Polybiidae | *Polybius* | *Polybius pusillus* | *Liocarcinus pusillus* (Leach, 1816) |
| Animalia | Arthropoda | Malacostraca | Decapoda | Polybiidae | *Polybius* | *Polybius vernalis* | *Liocarcinus vernalis* (Risso, 1827) |
| Animalia | Arthropoda | Malacostraca | Decapoda | Polybiidae | *Polybius* | *Polybius zariquieyi* | *Liocarcinus zariquieyi* (Gordon, 1968) |
| Animalia | Chordata | Ascidiacea | Aplousobranchia | Didemnidae | *Lissoclinum* | *Lissoclinum perforatum* | *Lissoclinum perforatum* (Giard, 1872) |
| Animalia | Mollusca | Bivalvia | Mytilida | Mytilidae | *Lithophaga* | *Lithophaga lithophaga* | *Lithophaga lithophaga* (Linnaeus, 1758) |
| Animalia | Annelida | Polychaeta | Eunicida | Lumbrineridae | *Scoletoma* | *Scoletoma tetraura* | *Lumbrineris tetraura* (Schmarda, 1861) |
| Animalia | Annelida | Polychaeta | Sabellida | Sabellariidae | *Lygdamis* | *Lygdamis muratus* | *Lygdamis muratus* (Allen, 1904) |
| Animalia | Mollusca | Gastropoda |  |  | *Lymnaea* | *Lymnaea stagnalis* | *Lymnaea stagnalis* (Linnaeus, 1758) |
| Animalia | Annelida | Polychaeta | Eunicida | Eunicidae | *Lysidice* | *Lysidice collaris* | *Lysidice collaris* Grube, 1868 |
| Animalia | Annelida | Polychaeta | Eunicida | Eunicidae | *Lysidice* | *Lysidice ninetta* | *Lysidice ninetta* Audouin & H Milne Edwards, 1833 |
| Animalia | Arthropoda | Malacostraca | Decapoda | Lysmatidae | *Lysmata* | *Lysmata seticaudata* | *Lysmata seticaudata* (Risso, 1816) |
| Animalia | Mollusca | Bivalvia | Cardiida | Tellinidae | *Macomangulus* | *Macomangulus tenuis* | *Macomangulus tenuis* (da Costa, 1778) |
| Animalia | Arthropoda | Malacostraca | Decapoda | Inachidae | *Macropodia* | *Macropodia rostrata* | *Macropodia rostrata* (Linnaeus, 1761) |
| Animalia | Mollusca | Bivalvia | Venerida | Mactridae | *Mactra* | *Mactra glauca* | *Mactra glauca* Born, 1778 |
| Animalia | Mollusca | Bivalvia | Venerida | Mactridae | *Mactra* | *Mactra stultorum* | *Mactra stultorum* (Linnaeus, 1758) |
| Animalia | Mollusca | Bivalvia | Ostreida | Ostreidae | *Magallana* | *Magallana gigas* | *Magallana gigas* (Thunberg, 1793) |
| Animalia | Annelida | Polychaeta |  | Magelonidae | *Magelona* | *Magelona alleni* | *Magelona alleni* Wilson, 1958 |
| Animalia | Annelida | Polychaeta |  | Magelonidae | *Magelona* | *Magelona minuta* | *Magelona minuta* Eliason, 1962 |
| Animalia | Arthropoda | Malacostraca | Decapoda | Majidae | *Maja* | *Maja crispata* | *Maja crispata* Risso, 1827 |
| Animalia | Annelida | Polychaeta |  | Maldanidae | *Maldane* | *Maldane glebifex* | *Maldane glebifex* Grube, 1860 |
| Animalia | Annelida | Polychaeta |  | Maldanidae | *Maldane* | *Maldane sarsi* | *Maldane sarsi* Malmgren, 1865 |
| Animalia | Annelida | Polychaeta | Phyllodocida | Polynoidae | *Malmgrenia* | *Malmgrenia lunulata* | *Malmgrenia lunulata* (Delle Chiaje, 1830) |
| Animalia | Mollusca | Gastropoda | Neogastropoda | Mangeliidae | *Villiersiella* | *Villiersiella attenuata* | *Mangelia attenuata* (Montagu, 1803) |
| Animalia | Annelida | Polychaeta |  | Capitellidae | *Mediomastus* | *Mediomastus capensis* | *Mediomastus capensis* Day, 1961 |
| Animalia | Arthropoda | Malacostraca | Decapoda | Dorippidae | *Medorippe* | *Medorippe lanata* | *Medorippe lanata* (Linnaeus, 1767) |
| Animalia | Arthropoda | Malacostraca | Amphipoda | Megaluropidae | *Megaluropus* | *Megaluropus agilis* | *Megaluropus agilis* Hoek, 1889 |
| Animalia | Annelida | Polychaeta |  | Melinnidae | *Melinna* | *Melinna palmata* | *Melinna palmata* Grube, 1870 |
| Animalia | Arthropoda | Malacostraca | Amphipoda | Melitidae | *Melita* | *Melita hergensis* | *Melita hergensis* Reid, 1939 |
| Animalia | Arthropoda | Malacostraca | Amphipoda | Melitidae | *Melita* | *Melita palmata* | *Melita palmata* (Montagu, 1804) |
| Animalia | Arthropoda | Malacostraca | Mysida | Mysidae | *Mesopodopsis* | *Mesopodopsis slabberi* | *Mesopodopsis slabberi* (Van Beneden, 1861) |
| Animalia | Arthropoda | Malacostraca | Amphipoda | Aoridae | *Microdeutopus* | *Microdeutopus anomalus* | *Microdeutopus anomalus* (Rathke, 1843) |
| Animalia | Bryozoa | Gymnolaemata | Cheilostomatida | Microporidae | *Micropora* | *Micropora coriacea* | *Micropora coriacea* (Johnston, 1847) |
| Animalia | Arthropoda | Malacostraca | Amphipoda | Microprotopidae | *Microprotopus* | *Microprotopus maculatus* | *Microprotopus maculatus* Norman, 1867 |
| Animalia | Mollusca | Bivalvia | Pectinida | Pectinidae | *Mimachlamys* | *Mimachlamys varia* | *Mimachlamys varia* (Linnaeus, 1758) |
| Animalia | Mollusca | Gastropoda | Neogastropoda | Columbellidae | *Mitrella* | *Mitrella scripta* | *Mitrella scripta* (Linnaeus, 1758) |
| Animalia | Mollusca | Bivalvia | Mytilida | Modiolidae | *Modiolus* | *Modiolus barbatus* | *Modiolus barbatus* (Linnaeus, 1758) |
| Animalia | Chordata | Ascidiacea | Stolidobranchia | Molgulidae | *Molgula* | *Molgula manhattensis* | *Molgula manhattensis* (De Kay, 1843) |
| Animalia | Arthropoda | Malacostraca | Amphipoda | Corophiidae | *Monocorophium* | *Monocorophium acherusicum* | *Monocorophium acherusicum* (A. Costa, 1853) |
| Animalia | Arthropoda | Malacostraca | Amphipoda | Corophiidae | *Monocorophium* | *Monocorophium insidiosum* | *Monocorophium insidiosum* (Crawford, 1937) |
| Animalia | Arthropoda | Malacostraca | Amphipoda | Corophiidae | *Monocorophium* | *Monocorophium sextonae* | *Monocorophium sextonae* (Crawford, 1937) |
| Animalia | Mollusca | Gastropoda | Neogastropoda | Muricidae | *Muricopsis* | *Muricopsis cristata* | *Muricopsis cristata* (Brocchi, 1814) |
| Animalia | Mollusca | Bivalvia | Mytilida | Mytilidae | *Musculus* | *Musculus costulatus* | *Musculus costulatus* (Risso, 1826) |
| Animalia | Mollusca | Bivalvia | Mytilida | Mytilidae | *Musculus* | *Musculus discors* | *Musculus discors* (Linnaeus, 1767) |
| Animalia | Mollusca | Bivalvia |  | Myidae | *Mya* | *Mya arenaria* | *Mya arenaria* Linnaeus, 1758 |
| Animalia | Annelida | Polychaeta | Phyllodocida | Syllidae | *Myrianida* | *Myrianida prolifera* | *Myrianida prolifera* (O.F. Müller, 1788) |
| Animalia | Mollusca | Bivalvia | Venerida | Veneridae | *Mysia* | *Mysia undata* | *Mysia undata* (Pennant, 1777) |
| Animalia | Mollusca | Bivalvia | Mytilida | Mytilidae | *Mytilus* | *Mytilus edulis* | *Mytilus edulis* Linnaeus, 1758 |
| Animalia | Mollusca | Gastropoda | Neogastropoda | Nassariidae | *Tritia* | *Tritia nitida* | *Nassarius nitidus* (Jeffreys, 1867) |
| Animalia | Mollusca | Gastropoda | Neogastropoda | Nassariidae | *Tritia* | *Tritia reticulata* | *Nassarius (Hinia)* reticulatus (Linnaeus, 1758) |
| Animalia | Arthropoda | Malacostraca | Isopoda | Cirolanidae | *Natatolana* | *Natatolana borealis* | *Natatolana borealis* (Liljeborg, 1851) |
| Animalia | Mollusca | Gastropoda | Littorinimorpha | Naticidae | *Naticarius* | *Naticarius stercusmuscarum* | *Naticarius stercusmuscarum* (Gmelin, 1791) |
| Animalia | Arthropoda | Malacostraca | Decapoda | Callianassidae | *Necallianassa* | *Necallianassa truncata* | *Necallianassa truncata* (Giard & Bonnier, 1890) |
| Animalia | Arthropoda | Malacostraca | Decapoda | Nephropidae | *Nephrops* | *Nephrops norvegicus* | *Nephrops norvegicus* (Linnaeus, 1758) |
| Animalia | Annelida | Polychaeta | Phyllodocida | Nephtyidae | *Nephtys* | *Nephtys cirrosa* | *Nephtys cirrosa* Ehlers, 1868 |
| Animalia | Annelida | Polychaeta | Phyllodocida | Nephtyidae | *Nephtys* | *Nephtys hombergii* | *Nephtys hombergii* Savigny in Lamarck, 1818 |
| Animalia | Annelida | Polychaeta | Phyllodocida | Nephtyidae | *Nephtys* | *Nephtys hystricis* | *Nephtys hystricis* McIntosh, 1900 |
| Animalia | Annelida | Polychaeta | Phyllodocida | Nephtyidae | *Nephtys* | *Nephtys incisa* | *Nephtys incisa* Malmgren, 1865 |
| Animalia | Annelida | Polychaeta | Phyllodocida | Phyllodocidae | *Nereiphylla* | *Nereiphylla rubiginosa* | *Nereiphylla rubiginosa* (Saint-Joseph, 1888) |
| Animalia | Annelida | Polychaeta | Phyllodocida | Nereididae | *Nereis* | *Nereis pelagica* | *Nereis pelagica* Linnaeus, 1758 |
| Animalia | Annelida | Polychaeta | Phyllodocida | Nereididae | *Nereis* | *Nereis splendida* | *Nereis splendida* Grube, 1840 |
| Animalia | Annelida | Polychaeta |  | Terebellidae | *Nicolea* | *Nicolea venustula* | *Nicolea venustula* (Montagu, 1819) |
| Animalia | Annelida | Polychaeta |  | Maldanidae | *Nicomache* | *Nicomache lumbricalis* | *Nicomache lumbricalis* (Fabricius, 1780) |
| Animalia | Mollusca | Gastropoda | Littorinimorpha | Anabathridae | *Nodulus* | *Nodulus contortus* | *Nodulus contortus* (Jeffreys, 1856) |
| Animalia | Bryozoa | Gymnolaemata | Ctenostomatida | Nolellidae | *Nolella* | *Nolella stipata* | *Nolella stipata* Gosse, 1855 |
| Animalia | Annelida | Polychaeta | Eunicida | Onuphidae | *Nothria* | *Nothria conchylega* | *Nothria conchylega* (Sars, 1835) |
| Animalia | Annelida | Polychaeta |  | Capitellidae | *Notomastus* | *Notomastus latericeus* | *Notomastus latericeus* M. Sars, 1851 |
| Animalia | Mollusca | Bivalvia | Nuculida | Nuculidae | *Nucula* | *Nucula nitidosa* | *Nucula nitidosa* Winckworth, 1930 |
| Animalia | Mollusca | Bivalvia | Nuculida | Nuculidae | *Nucula* | *Nucula nucleus* | *Nucula nucleus* (Linnaeus, 1758) |
| Animalia | Mollusca | Bivalvia | Nuculida | Nuculidae | *Nucula* | *Nucula sulcata* | *Nucula sulcata* Bronn, 1831 |
| Animalia | Cnidaria | Hydrozoa | Leptothecata | Campanulariidae | *Obelia* | *Obelia bidentata* | *Obelia bidentata* Clark, 1875 |
| Animalia | Cnidaria | Hydrozoa | Leptothecata | Campanulariidae | *Obelia* | *Obelia dichotoma* | *Obelia dichotoma* (Linnaeus, 1758) |
| Animalia | Cnidaria | Hydrozoa | Leptothecata | Campanulariidae | *Obelia* | *Obelia geniculata* | *Obelia geniculata* (Linnaeus, 1758) |
| Animalia | Cnidaria | Hydrozoa | Leptothecata | Campanulariidae | *Obelia* | *Obelia longissima* | *Obelia longissima* (Pallas, 1766) |
| Animalia | Mollusca | Gastropoda | Neogastropoda | Muricidae | *Ocenebra* | *Ocenebra edwardsii* | *Ocenebra edwardsii* (Payraudeau, 1826) |
| Animalia | Mollusca | Gastropoda | Neogastropoda | Muricidae | *Ocinebrina* | *Ocinebrina aciculata* | *Ocinebrina aciculata* (Lamarck, 1822) |
| Animalia | Annelida | Polychaeta | Phyllodocida | Syllidae | *Odontosyllis* | *Odontosyllis gibba* | *Odontosyllis gibba* Claparède, 1863 |
| Animalia | Mollusca | Gastropoda |  | Pyramidellidae | *Odostomia* | *Odostomia plicata* | *Odostomia plicata* (Montagu, 1803) |
| Animalia | Echinodermata | Holothuroidea | Apodida | Synaptidae | *Oestergrenia* | *Oestergrenia digitata* | *Oestergrenia digitata* (Montagu, 1815) |
| Animalia | Annelida | Polychaeta | Eunicida | Onuphidae | *Onuphis* | *Onuphis eremita* | *Onuphis eremita* Audouin & Milne Edwards, 1833 |
| Animalia | Echinodermata | Ophiuroidea | Amphilepidida | Ophiotrichidae | *Ophiothrix* | *Ophiothrix fragilis* | *Ophiothrix fragilis* (Abildgaard in O.F. Müller, 1789) |
| Animalia | Echinodermata | Ophiuroidea | Ophiurida | Ophiuridae | *Ophiura* | *Ophiura albida* | *Ophiura albida* Forbes, 1839 |
| Animalia | Echinodermata | Ophiuroidea | Ophiurida | Ophiuridae | *Ophiura* | *Ophiura ophiura* | *Ophiura ophiura* (Linnaeus, 1758) |
| Animalia | Annelida | Polychaeta |  | Orbiniidae | *Orbinia* | *Orbinia sertulata* | *Orbinia sertulata* (Savigny, 1822) |
| Animalia | Arthropoda | Malacostraca | Amphipoda | Tryphosidae | *Orchomene* | *Orchomene humilis* | *Orchomene humilis* (A. Costa, 1853) |
| Animalia | Mollusca | Bivalvia | Ostreida | Ostreidae | *Ostrea* | *Ostrea edulis* | *Ostrea edulis* Linnaeus, 1758 |
| Animalia | Echinodermata | Echinoidea | Spatangoida | Schizasteridae | *Ova* | *Ova canalifera* | *Ova canalifera* (Lamarck, 1816) |
| Animalia | Annelida | Polychaeta | Sabellida | Oweniidae | *Owenia* | *Owenia fusiformis* | *Owenia fusiformis* Delle Chiaje, 1844 |
| Animalia | Arthropoda | Malacostraca | Decapoda | Grapsidae | *Pachygrapsus* | *Pachygrapsus marmoratus* | *Pachygrapsus marmoratus* (Fabricius, 1787) |
| Animalia | Arthropoda | Malacostraca | Decapoda | Diogenidae | *Paguristes* | *Paguristes eremita* | *Paguristes eremita* (Linnaeus, 1767) |
| Animalia | Arthropoda | Malacostraca | Decapoda | Paguridae | *Pagurus* | *Pagurus anachoretus* | *Pagurus anachoretus* Risso, 1827 |
| Animalia | Arthropoda | Malacostraca | Decapoda | Paguridae | *Pagurus* | *Pagurus bernhardus* | *Pagurus bernhardus* (Linnaeus, 1758) |
| Animalia | Arthropoda | Malacostraca | Decapoda | Paguridae | *Pagurus* | *Pagurus cuanensis* | *Pagurus cuanensis* Bell, 1846 |
| Animalia | Arthropoda | Malacostraca | Decapoda | Paguridae | *Pagurus* | *Pagurus excavatus* | *Pagurus excavatus* (J. F. W. Herbst, 1791) |
| Animalia | Arthropoda | Malacostraca | Decapoda | Paguridae | *Pagurus* | *Pagurus prideaux* | *Pagurus prideaux* Leach, 1815 |
| Animalia | Arthropoda | Malacostraca | Decapoda | Palaemonidae | *Palaemon* | *Palaemon adspersus* | *Palaemon adspersus* Rathke, 1837 |
| Animalia | Arthropoda | Malacostraca | Decapoda | Palaemonidae | *Palaemon* | *Palaemon antennarius* | *Palaemon antennarius* H. Milne-Edwards, 1837 |
| Animalia | Arthropoda | Malacostraca | Decapoda | Palaemonidae | *Palaemon* | *Palaemon serratus* | *Palaemon serratus* (Pennant, 1777) |
| Animalia | Arthropoda | Malacostraca | Decapoda | Palicidae | *Palicus* | *Palicus caronii* | *Palicus caronii* (Roux, 1830) |
| Animalia | Mollusca | Gastropoda |  | Polyceridae | *Palio* | *Palio dubia* | *Palio dubia* (M. Sars, 1829) |
| Animalia | Annelida | Polychaeta | Eunicida | Eunicidae | *Palola* | *Palola siciliensis* | *Palola siciliensis* (Grube, 1840) |
| Animalia | Mollusca | Bivalvia | Cardiida | Cardiidae | *Papillicardium* | *Papillicardium papillosum* | *Papillicardium papillosum* (Poli, 1791) |
| Animalia | Echinodermata | Echinoidea |  | Parechinidae | *Paracentrotus* | *Paracentrotus lividus* | *Paracentrotus lividus* (Lamarck, 1816) |
| Animalia | Annelida | Polychaeta |  | Paraonidae | *Paradoneis* | *Paradoneis armata* | *Paradoneis armata* Glémarec, 1966 |
| Animalia | Annelida | Polychaeta |  | Paraonidae | *Paradoneis* | *Paradoneis ilvana* | *Paradoneis ilvana* Castelli, 1985 |
| Animalia | Annelida | Polychaeta |  | Paraonidae | *Paradoneis* | *Paradoneis lyra* | *Paradoneis lyra* (Southern, 1914) |
| Animalia | Mollusca | Gastropoda |  | Discodorididae | *Paradoris* | *Paradoris indecora* | *Paradoris indecora* (Bergh, 1881) |
| Animalia | Echinodermata | Holothuroidea | Dendrochirotida | Cucumariidae | *Paraleptopentacta* | *Paraleptopentacta elongata* | *Paraleptopentacta elongata* (Düben & Koren, 1846) |
| Animalia | Annelida | Polychaeta |  | Paraonidae | *Paraonis* | *Paraonis fulgens* | *Paraonis fulgens* (Levinsen, 1884) |
| Animalia | Arthropoda | Malacostraca | Amphipoda | Caprellidae | *Pariambus* | *Pariambus typicus* | *Pariambus typicus* (Krøyer, 1845) |
| Animalia | Arthropoda | Malacostraca | Decapoda | Parthenopidae | *Parthenopoides* | *Parthenopoides massena* | *Parthenopoides massena* (Roux, 1830) |
| Animalia | Mollusca | Bivalvia | Cardiida | Cardiidae | *Parvicardium* | *Parvicardium exiguum* | *Parvicardium exiguum* (Gmelin, 1791) |
| Animalia | Mollusca | Bivalvia | Cardiida | Cardiidae | *Papillicardium* | *Papillicardium minimum* | *Parvicardium minimum* (R. A. Philippi, 1836) |
| Animalia | Mollusca | Bivalvia | Cardiida | Cardiidae | *Parvicardium* | *Parvicardium pinnulatum* | *Parvicardium pinnulatum* (Conrad, 1831) |
| Animalia | Mollusca | Gastropoda | Patellida | Patellidae | *Patella* | *Patella caerulea* | *Patella caerulea* Linnaeus, 1758 |
| Animalia | Annelida | Polychaeta | Eunicida | Eunicidae | *Paucibranchia* | *Paucibranchia bellii* | *Paucibranchia bellii* (Audouin & Milne Edwards, 1833) |
| Animalia | Arthropoda | Malacostraca | Amphipoda | Gammaridae | *Pectenogammarus* | *Pectenogammarus olivii* | *Pectenogammarus olivii* (H. Milne Edwards, 1830) |
| Animalia | Entoprocta |  | Solitaria | Pedicellinidae | *Pedicellina* | *Pedicellina cernua* | *Pedicellina cernua* (Pallas, 1774) |
| Animalia | Arthropoda | Malacostraca | Decapoda | Penaeidae | *Penaeus* | *Penaeus kerathurus* | *Penaeus kerathurus* (Forskål, 1775) |
| Animalia | Arthropoda | Thecostraca | Balanomorpha | Balanidae | *Perforatus* | *Perforatus perforatus* | *Perforatus perforatus* (Bruguière, 1789) |
| Animalia | Annelida | Polychaeta | Phyllodocida | Nereididae | *Perinereis* | *Perinereis cultrifera* | *Perinereis cultrifera* (Grube, 1840) |
| Animalia | Mollusca | Gastropoda | Littorinimorpha | Hydrobiidae | *Peringia* | *Peringia ulvae* | *Peringia ulvae* (Pennant, 1777) |
| Animalia | Sipuncula | Phascolosomatidea | Phascolosomatiformes | Phascolosomatidae | *Phascolosoma* | *Phascolosoma granulatum* | *Phascolosoma (Phascolosoma)* granulatum Leuckart, 1828 |
| Animalia | Mollusca | Bivalvia | Adapedonta | Pharidae | *Phaxas* | *Phaxas pellucidus* | *Phaxas pellucidus* (Pennant, 1777) |
| Animalia | Annelida | Polychaeta |  | Flabelligeridae | *Pherusa* | *Pherusa plumosa* | *Pherusa plumosa* (Müller, 1776) |
| Animalia | Mollusca | Gastropoda |  | Philinidae | *Philine* | *Philine aperta* | *Philine aperta* (Linnaeus, 1767) |
| Animalia | Mollusca | Gastropoda |  | Philinidae | *Philine* | *Philine catena* | *Philine catena* (Montagu, 1803) |
| Animalia | Mollusca | Gastropoda |  | Aglajidae | *Philinopsis* | *Philinopsis depicta* | *Philinopsis depicta* (Renier, 1807) |
| Animalia | Arthropoda | Malacostraca | Decapoda | Crangonidae | *Philocheras* | *Philocheras bispinosus* | *Philocheras bispinosus* (Hailstone, 1835) |
| Animalia | Arthropoda | Malacostraca | Decapoda | Crangonidae | *Philocheras* | *Philocheras trispinosus* | *Philocheras trispinosus* (Hailstone in Hailstone & Westwood, 1835) |
| Animalia | Mollusca | Bivalvia |  | Pholadidae | *Pholas* | *Pholas dactylus* | *Pholas dactylus* Linnaeus, 1758 |
| Animalia | Annelida | Polychaeta | Phyllodocida | Sigalionidae | *Pholoe* | *Pholoe minuta* | *Pholoe minuta* (Fabricius, 1780) |
| Animalia | Mollusca | Gastropoda | Trochida | Trochidae | *Phorcus* | *Phorcus richardi* | *Phorcus richardi* (Payraudeau, 1826) |
| Animalia | Phoronida |  |  | Phoronidae | *Phoronis* | *Phoronis muelleri* | *Phoronis muelleri* Selys-Lonchamps, 1903 |
| Animalia | Phoronida |  |  | Phoronidae | *Phoronis* | *Phoronis psammophila* | *Phoronis psammophila* Cori, 1889 |
| Animalia | Arthropoda | Malacostraca | Amphipoda | Photidae | *Photis* | *Photis longicaudata* | *Photis longicaudata* (Spence Bate & Westwood, 1862) |
| Animalia | Arthropoda | Malacostraca | Amphipoda | Caprellidae | *Phtisica* | *Phtisica marina* | *Phtisica marina* Slabber, 1769 |
| Animalia | Annelida | Polychaeta | Phyllodocida | Phyllodocidae | *Phyllodoce* | *Phyllodoce groenlandica* | *Phyllodoce groenlandica* Örsted, 1842 |
| Animalia | Annelida | Polychaeta | Phyllodocida | Phyllodocidae | *Phyllodoce* | *Phyllodoce laminosa* | *Phyllodoce laminosa* Lamarck, 1818 (from Savigny Mss) |
| Animalia | Annelida | Polychaeta | Phyllodocida | Phyllodocidae | *Phyllodoce* | *Phyllodoce lineata* | *Phyllodoce lineata* (Claparède, 1870) |
| Animalia | Annelida | Polychaeta | Phyllodocida | Phyllodocidae | *Phyllodoce* | *Phyllodoce mucosa* | *Phyllodoce mucosa* Örsted, 1843 |
| Animalia | Annelida | Polychaeta | Phyllodocida | Phyllodocidae | *Phyllodoce* | *Phyllodoce schmardaei* | *Phyllodoce schmardaei* Day, 1963 |
| Animalia | Annelida | Polychaeta |  | Orbiniidae | *Phylo* | *Phylo foetida* | *Phylo foetida* (Claparède, 1868) |
| Animalia | Mollusca | Gastropoda |  | Physidae | *Physella* | *Physella acuta* | *Physella acuta* (Draparnaud, 1805) |
| Animalia | Annelida | Polychaeta | Phyllodocida | Pilargidae | *Pilargis* | *Pilargis verrucosa* | *Pilargis verrucosa* Saint-Joseph, 1899 |
| Animalia | Arthropoda | Malacostraca | Decapoda | Pilumnidae | *Pilumnus* | *Pilumnus hirtellus* | *Pilumnus hirtellus* (Linnaeus, 1761) |
| Animalia | Mollusca | Bivalvia | Ostreida | Pinnidae | *Pinna* | *Pinna nobilis* | *Pinna nobilis* Linnaeus, 1758 |
| Animalia | Arthropoda | Malacostraca | Decapoda | Pinnotheridae | *Pinnotheres* | *Pinnotheres pisum* | *Pinnotheres pisum* (Linnaeus, 1767) |
| Animalia | Arthropoda | Malacostraca | Decapoda | Epialtidae | *Pisa* | *Pisa armata* | *Pisa armata* (Latreille, 1803) |
| Animalia | Arthropoda | Malacostraca | Decapoda | Epialtidae | *Pisa* | *Pisa nodipes* | *Pisa nodipes* Leach, 1815 |
| Animalia | Mollusca | Gastropoda | Neogastropoda | Pisaniidae | *Pisania* | *Pisania striata* | *Pisania striata* (Gmelin, 1791) |
| Animalia | Arthropoda | Malacostraca | Decapoda | Porcellanidae | *Pisidia* | *Pisidia bluteli* | *Pisidia bluteli* (Risso, 1816) |
| Animalia | Arthropoda | Malacostraca | Decapoda | Porcellanidae | *Pisidia* | *Pisidia longicornis* | *Pisidia longicornis* (Linnaeus, 1767) |
| Animalia | Mollusca | Gastropoda | Littorinimorpha | Anabathridae | *Pisinna* | *Pisinna glabrata* | *Pisinna glabrata* (Megerle von Mühlfeld, 1824) |
| Animalia | Annelida | Polychaeta |  | Terebellidae | *Pista* | *Pista cristata* | *Pista cristata* (Müller, 1776) |
| Animalia | Bryozoa | Stenolaemata | Cyclostomatida | Plagioeciidae | *Plagioecia* | *Plagioecia patina* | *Plagioecia patina* (Lamarck, 1816) |
| Animalia | Mollusca | Gastropoda |  | Discodorididae | *Platydoris* | *Platydoris argo* | *Platydoris argo* (Linnaeus, 1767) |
| Animalia | Annelida | Polychaeta | Phyllodocida | Nereididae | *Platynereis* | *Platynereis dumerilii* | *Platynereis dumerilii* (Audouin & Milne Edwards, 1833) |
| Animalia | Mollusca | Gastropoda | Pleurobranchida | Pleurobranchidae | *Pleurehdera* | *Pleurehdera stellata* | *Pleurehdera stellata* (Risso, 1826) |
| Animalia | Annelida | Polychaeta | Phyllodocida | Hesionidae | *Podarkeopsis* | *Podarkeopsis capensis* | *Podarkeopsis capensis* (Day, 1963) |
| Animalia | Annelida | Polychaeta |  | Poecilochaetidae | *Poecilochaetus* | *Poecilochaetus serpens* | *Poecilochaetus serpens* Allen, 1904 |
| Animalia | Mollusca | Bivalvia | Venerida | Veneridae | *Polititapes* | *Polititapes aureus* | *Polititapes aureus* (Gmelin, 1791) |
| Animalia | Mollusca | Bivalvia | Venerida | Veneridae | *Polititapes* | *Polititapes rhomboides* | *Polititapes rhomboides* (Pennant, 1777) |
| Animalia | Annelida | Polychaeta |  | Terebellidae | *Polycirrus* | *Polycirrus aurantiacus* | *Polycirrus aurantiacus* Grube, 1860 |
| Animalia | Arthropoda | Malacostraca | Decapoda | Carcinidae | *Portumnus* | *Portumnus latipes* | *Portumnus latipes* (Pennant, 1777) |
| Animalia | Arthropoda | Malacostraca | Decapoda | Potamidae | *Potamon* | *Potamon fluviatile* | *Potamon fluviatile* (Herbst, 1785) |
| Animalia | Annelida | Polychaeta |  | Maldanidae | *Praxillella* | *Praxillella affinis* | *Praxillella affinis* (M. Sars in G.O. Sars, 1872) |
| Animalia | Annelida | Polychaeta |  | Maldanidae | *Praxillella* | *Praxillella gracilis* | *Praxillella gracilis* (M. Sars, 1861) |
| Animalia | Annelida | Polychaeta |  | Spionidae | *Prionospio* | *Prionospio cirrifera* | *Prionospio cirrifera* Wirén, 1883 |
| Animalia | Annelida | Polychaeta |  | Spionidae | *Prionospio* | *Prionospio fallax* | *Prionospio fallax* Söderström, 1920 |
| Animalia | Annelida | Polychaeta |  | Spionidae | *Prionospio* | *Prionospio malmgreni* | *Prionospio malmgreni* Claparede |
| Animalia | Arthropoda | Malacostraca | Decapoda | Processidae | *Processa* | *Processa edulis* | *Processa edulis* (Risso, 1816) |
| Animalia | Annelida | Polychaeta | Eunicida | Dorvilleidae | *Protodorvillea* | *Protodorvillea kefersteini* | *Protodorvillea kefersteini* (McIntosh, 1869) |
| Animalia | Arthropoda | Malacostraca | Cumacea | Pseudocumatidae | *Pseudocuma* | *Pseudocuma longicorne* | *Pseudocuma (Pseudocuma)* longicorne (Bate, 1858) |
| Animalia | Annelida | Polychaeta | Phyllodocida | Phyllodocidae | *Pseudomystides* | *Pseudomystides limbata* | *Pseudomystides limbata* (Saint-Joseph, 1888) |
| Animalia | Annelida | Polychaeta | Sabellida | Sabellidae | *Pseudopotamilla* | *Pseudopotamilla reniformis* | *Pseudopotamilla reniformis* (Bruguière, 1789) |
| Animalia | Arthropoda | Malacostraca | Amphipoda | Caprellidae | *Pseudoprotella* | *Pseudoprotella phasma* | *Pseudoprotella phasma* (Montagu, 1804) |
| Animalia | Echinodermata | Holothuroidea | Dendrochirotida | Sclerodactylidae | *Pseudothyone* | *Pseudothyone raphanus* | *Pseudothyone raphanus* (Düben & Koren, 1846) |
| Animalia | Annelida | Polychaeta | Phyllodocida | Phyllodocidae | *Pterocirrus* | *Pterocirrus macroceros* | *Pterocirrus macroceros* (Grube, 1860) |
| Animalia | Mollusca | Gastropoda | Littorinimorpha | Rissoidae | *Pusillina* | *Pusillina sarsii* | **Pusillina sarsi** |
| Animalia | Arthropoda | Malacostraca | Amphipoda | Maeridae | *Quadrimaera* | *Quadrimaera inaequipes* | *Quadrimaera inaequipes* (A. Costa in Hope, 1851) |
| Animalia | Mollusca | Gastropoda |  |  | *Peregriana* | *Peregriana peregra* | *Radix (Radix)* peregra (O. F. Müller, 1774) |
| Animalia | Mollusca | Gastropoda | Neogastropoda | Muricidae | *Rapana* | *Rapana venosa* | *Rapana venosa* (Valenciennes, 1846) |
| Animalia | Mollusca | Gastropoda | Neogastropoda | Raphitomidae | *Raphitoma* | *Raphitoma bicolor* | *Raphitoma bicolor* (Risso, 1826) |
| Animalia | Mollusca | Gastropoda | Neogastropoda | Raphitomidae | *Raphitoma* | *Raphitoma purpurea* | *Raphitoma purpurea* (Montagu, 1803) |
| Animalia | Bryozoa | Gymnolaemata | Cheilostomatida | Adeonidae | *Reptadeonella* | *Reptadeonella violacea* | *Reptadeonella violacea* (Johnston, 1847) |
| Animalia | Bryozoa | Gymnolaemata | Cheilostomatida | Phidoloporidae | *Reteporella* | *Reteporella beaniana* | *Reteporella beaniana* (King, 1846) |
| Animalia | Mollusca | Gastropoda |  | Retusidae | *Retusa* | *Retusa truncatula* | *Retusa truncatula* (Bruguière, 1792) |
| Animalia | Arthropoda | Malacostraca | Decapoda | Panopeidae | *Rhithropanopeus* | *Rhithropanopeus harrisii* | *Rhithropanopeus harrisii* (Gould, 1841) |
| Animalia | Mollusca | Gastropoda | Littorinimorpha | Rissoidae | *Rissoa* | *Rissoa parva* | *Rissoa parva* (da Costa, 1778) |
| Animalia | Mollusca | Gastropoda |  | Rissoellidae | *Rissoella* | *Rissoella diaphana* | *Rissoella diaphana* (Alder, 1848) |
| Animalia | Mollusca | Bivalvia | Venerida | Veneridae | *Ruditapes* | *Ruditapes philippinarum* | *Ruditapes philippinarum* (A. Adams & Reeve, 1850) |
| Animalia | Annelida | Polychaeta | Sabellida | Sabellidae | *Sabella* | *Sabella pavonina* | *Sabella pavonina* Savigny, 1822 |
| Animalia | Annelida | Polychaeta | Sabellida | Sabellariidae | *Sabellaria* | *Sabellaria spinulosa* | *Sabellaria spinulosa* (Leuckart, 1849) |
| Animalia | Mollusca | Bivalvia | Ostreida | Ostreidae | *Saccostrea* | *Saccostrea glomerata* | *Saccostrea glomerata* (A. A. Gould, 1850) |
| Animalia | Mollusca | Gastropoda |  | Scaphandridae | *Scaphander* | *Scaphander lignarius* | *Scaphander lignarius* (Linnaeus, 1758) |
| Animalia | Bryozoa | Gymnolaemata | Cheilostomatida | Schizoporellidae | *Schizoporella* | *Schizoporella errata* | *Schizoporella errata* (Waters, 1878) |
| Animalia | Annelida | Polychaeta |  | Spionidae | *Scolelepis* | *Scolelepis bonnieri* | *Scolelepis bonnieri* (Mesnil, 1896) |
| Animalia | Annelida | Polychaeta |  | Orbiniidae | *Scoloplos* | *Scoloplos armiger* | *Scoloplos armiger* (Müller, 1776) |
| Animalia | Bryozoa | Gymnolaemata | Cheilostomatida | Candidae | *Scrupocellaria* | *Scrupocellaria scruposa* | *Scrupocellaria scruposa* (Linnaeus, 1758) |
| Animalia | Annelida | Polychaeta |  | Capitellidae | *Scyphoproctus* | *Scyphoproctus armatus* | *Scyphoproctus armatus* (Fauvel, 1929) |
| Animalia | Mollusca | Cephalopoda | Sepiolida | Sepiolidae | *Sepiola* | *Sepiola rondeletii* | *Sepiola rondeleti* Leach, 1817 |
| Animalia | Annelida | Polychaeta | Sabellida | Serpulidae | *Serpula* | *Serpula vermicularis* | *Serpula vermicularis* Linnaeus, 1767 |
| Animalia | Mollusca | Gastropoda | Littorinimorpha | Rissoidae | *Setia* | *Setia turriculata* | *Setia turriculata* Monterosato, 1884 |
| Animalia | Arthropoda | Malacostraca | Decapoda | Sicyoniidae | *Sicyonia* | *Sicyonia carinata* | *Sicyonia carinata* (Brünnich, 1768) |
| Animalia | Annelida | Polychaeta | Phyllodocida | Sigalionidae | *Sigalion* | *Sigalion mathildae* | *Sigalion mathildae* Audouin & Milne Edwards, 1832 |
| Animalia | Arthropoda | Malacostraca | Mysida | Mysidae | *Siriella* | *Siriella armata* | *Siriella armata* (Milne Edwards, 1837) |
| Animalia | Mollusca | Gastropoda | Littorinimorpha | Skeneopsidae | *Skeneopsis* | *Skeneopsis planorbis* | *Skeneopsis planorbis* (O. Fabricius, 1780) |
| Animalia | Mollusca | Bivalvia | Adapedonta | Solenidae | *Solen* | *Solen marginatus* | *Solen marginatus* Pulteney, 1799 |
| Animalia | Echinodermata | Echinoidea | Spatangoida | Spatangidae | *Spatangus* | *Spatangus purpureus* | *Spatangus purpureus* O.F. Müller, 1776 |
| Animalia | Echinodermata | Echinoidea |  | Toxopneustidae | *Sphaerechinus* | *Sphaerechinus granularis* | *Sphaerechinus granularis* (Lamarck, 1816) |
| Animalia | Arthropoda | Malacostraca | Isopoda | Sphaeromatidae | *Sphaeroma* | *Sphaeroma serratum* | *Sphaeroma serratum* (J. C. Fabricius, 1787) |
| Animalia | Annelida | Polychaeta |  | Spionidae | *Spio* | *Spio decorata* | *Spio decorata* Bobretzky, 1870 |
| Animalia | Annelida | Polychaeta |  | Chaetopteridae | *Spiochaetopterus* | *Spiochaetopterus costarum* | *Spiochaetopterus costarum* (Claparède, 1869) |
| Animalia | Annelida | Polychaeta |  | Spionidae | *Spiophanes* | *Spiophanes bombyx* | *Spiophanes bombyx* (Claparède, 1870) |
| Animalia | Annelida | Polychaeta |  | Spionidae | *Spiophanes* | *Spiophanes kroyeri* | *Spiophanes kroyeri* Grube, 1860 |
| Animalia | Mollusca | Bivalvia | Venerida | Mactridae | *Spisula* | *Spisula subtruncata* | *Spisula subtruncata* (da Costa, 1778) |
| Animalia | Mollusca | Gastropoda | Nudibranchia | Aeolidiidae | *Spurilla* | *Spurilla neapolitana* | *Spurilla neapolitana* (Delle Chiaje, 1841) |
| Animalia | Arthropoda | Malacostraca | Amphipoda | Stenothoidae | *Stenothoe* | *Stenothoe monoculoides* | *Stenothoe monoculoides* (Montagu, 1813) |
| Animalia | Arthropoda | Malacostraca | Amphipoda | Stenothoidae | *Stenothoe* | *Stenothoe valida* | *Stenothoe valida* Dana, 1853 |
| Animalia | Mollusca | Gastropoda | Trochida | Trochidae | *Steromphala* | *Steromphala adriatica* | *Steromphala adriatica* (R. A. Philippi, 1844) |
| Animalia | Mollusca | Gastropoda | Trochida | Trochidae | *Steromphala* | *Steromphala rarilineata* | *Steromphala rarilineata* (Michaud, 1829) |
| Animalia | Annelida | Polychaeta | Phyllodocida | Sigalionidae | *Sthenelais* | *Sthenelais boa* | *Sthenelais boa* (Johnston, 1833) |
| Animalia | Annelida | Polychaeta | Phyllodocida | Sigalionidae | *Sthenelais* | *Sthenelais limicola* | *Sthenelais limicola* (Ehlers, 1864) |
| Animalia | Mollusca | Gastropoda | Neogastropoda | Muricidae | *Stramonita* | *Stramonita haemastoma* | *Stramonita haemastoma* (Linnaeus, 1767) |
| Animalia | Annelida | Polychaeta |  | Spionidae | *Streblospio* | *Streblospio shrubsolii* | *Streblospio shrubsolii* (Buchanan, 1890) |
| Animalia | Mollusca | Bivalvia | Arcida | Noetiidae | *Striarca* | *Striarca lactea* | *Striarca lactea* (Linnaeus, 1758) |
| Animalia | Chordata | Ascidiacea | Stolidobranchia | Styelidae | *Styela* | *Styela plicata* | *Styela plicata* (Lesueur, 1823) |
| Animalia | Annelida | Polychaeta | Phyllodocida | Syllidae | *Syllis* | *Syllis gracilis* | *Syllis gracilis* Grube, 1840 |
| Animalia | Annelida | Polychaeta | Phyllodocida | Syllidae | *Syllis* | *Syllis hyalina* | *Syllis hyalina* Grube, 1863 |
| Animalia | Annelida | Polychaeta | Phyllodocida | Syllidae | *Syllis* | *Syllis variegata* | *Syllis variegata* Grube, 1860 |
| Animalia | Arthropoda | Malacostraca | Isopoda | Idoteidae | *Stenosoma* | *Stenosoma acuminatum* | *Synisoma acuminatum* (Leach, 1814) |
| Animalia | Mollusca | Bivalvia |  |  | *Tellimya* | *Tellimya ferruginosa* | *Tellimya ferruginosa* (Montagu, 1808) |
| Animalia | Annelida | Polychaeta |  | Terebellidae | *Terebella* | *Terebella lapidaria* | *Terebella lapidaria* Linnaeus, 1767 |
| Animalia | Annelida | Polychaeta |  | Trichobranchidae | *Terebellides* | *Terebellides stroemii* | *Terebellides stroemii* Sars, 1835 |
| Animalia | Nematoda | Chromadorea | Monhysterida | Linhomoeidae | *Terschellingia* | *Terschellingia longicaudata* | *Terschellingia longicaudata* de Man, 1907 |
| Animalia | Annelida | Polychaeta |  | Cirratulidae | *Tharyx* | *Tharyx killariensis* | *Tharyx killariensis* (Southern, 1914) |
| Animalia | Annelida | Polychaeta |  | Terebellidae | *Thelepus* | *Thelepus cincinnatus* | *Thelepus cincinnatus* (Fabricius, 1780) |
| Animalia | Annelida | Polychaeta |  | Orbiniidae | *Theodisca* |  | *Theodisca laevigata* (Grube, 1855) |
| Animalia | Annelida | Polychaeta |  | Orbiniidae | *Theodisca* |  | *Protoaricia oerstedii (Claparède,* 1864) |
| Animalia | Mollusca | Gastropoda | Cycloneritida | Neritidae | *Theodoxus* | *Theodoxus fluviatilis* | *Theodoxus fluviatilis* (Linnaeus, 1758) |
| Animalia | Arthropoda | Malacostraca | Decapoda | Thiidae | *Thia* | *Thia scutellata* | *Thia scutellata* (Fabricius, 1793) |
| Animalia | Arthropoda | Malacostraca | Decapoda | Thoridae | *Eualus* | *Eualus cranchii* | *Thoralus cranchii* (Leach, 1817) |
| Animalia | Mollusca | Bivalvia |  | Thraciidae | *Thracia* | *Thracia phaseolina* | *Thracia phaseolina* (Lamarck, 1818) |
| Animalia | Mollusca | Bivalvia | Lucinida | Thyasiridae | *Thyasira* | *Thyasira flexuosa* | *Thyasira flexuosa* (Montagu, 1803) |
| Animalia | Echinodermata | Holothuroidea | Dendrochirotida | Phyllophoridae | *Thyone* | *Thyone fusus* | *Thyone fusus* (O.F. Müller, 1776) |
| Animalia | Arthropoda | Malacostraca | Euphausiacea | Euphausiidae | *Thysanoessa* | *Thysanoessa inermis* | *Thysanoessa inermis* (Krøyer, 1846) |
| Animalia | Bryozoa | Gymnolaemata | Cheilostomatida | Candidae | *Tricellaria* | *Tricellaria inopinata* | *Tricellaria inopinata* d'Hondt & Occhipinti Ambrogi, 1985 |
| Animalia | Mollusca | Gastropoda | Trochida | Phasianellidae | *Tricolia* | *Tricolia pullus* | *Tricolia pullus* (Linnaeus, 1758) |
| Animalia | Mollusca | Gastropoda | Trochida | Phasianellidae | *Tricolia* | *Tricolia speciosa* | *Tricolia speciosa* (Megerle von Mühlfeld, 1824) |
| Animalia | Mollusca | Gastropoda | Trochida | Phasianellidae | *Tricolia* | *Tricolia tenuis* | *Tricolia tenuis* (Michaud, 1829) |
| Animalia | Mollusca | Gastropoda | Neogastropoda | Nassariidae | *Tritia* | *Tritia corniculum* | *Tritia corniculum* (Olivi, 1792) |
| Animalia | Mollusca | Gastropoda | Neogastropoda | Nassariidae | *Tritia* | *Tritia corrugata* | *Tritia cuvierii* (Payraudeau, 1826) |
| Animalia | Mollusca | Gastropoda | Neogastropoda | Nassariidae | *Tritia* | *Tritia incrassata* | *Tritia incrassata* (Strøm, 1768) |
| Animalia | Mollusca | Gastropoda | Neogastropoda | Nassariidae | *Tritia* | *Tritia mutabilis* | *Tritia mutabilis* (Linnaeus, 1758) |
| Animalia | Mollusca | Gastropoda | Neogastropoda | Nassariidae | *Tritia* | *Tritia neritea* | *Tritia neritea* (Linnaeus, 1758) |
| Animalia | Mollusca | Gastropoda | Neogastropoda | Nassariidae | *Tritia* | *Tritia pygmaea* | *Tritia pygmaea* (Schlotheim, 1820) |
| Animalia | Mollusca | Gastropoda | Neogastropoda | Muricidae | *Trophonopsis* | *Trophonopsis muricata* | *Trophonopsis muricata* (Montagu, 1803) |
| Animalia | Mollusca | Gastropoda | Littorinimorpha | Truncatellidae | *Truncatella* | *Truncatella subcylindrica* | *Truncatella subcylindrica* (Linnaeus, 1767) |
| Animalia | Annelida | Polychaeta | Phyllodocida | Syllidae | *Trypanosyllis* | *Trypanosyllis zebra* | *Trypanosyllis zebra* (Grube, 1860) |
| Animalia | Mollusca | Gastropoda |  | Pyramidellidae | *Pyrgisculus* | *Pyrgisculus jeffreysii* | *Turbonilla (Pyrgiscus)* jeffreysii (Jeffreys, 1848) |
| Animalia | Mollusca | Gastropoda |  | Turritellidae | *Turritellinella* | *Turritellinella tricarinata* | *Turritellinella tricarinata* (Brocchi, 1814) |
| Animalia | Arthropoda | Malacostraca | Isopoda | Tylidae | *Tylos* | *Tylos ponticus* | *Tylos ponticus* Grebnicki, 1874 |
| Animalia | Arthropoda | Malacostraca | Decapoda | Upogebiidae | *Upogebia* | *Upogebia deltaura* | *Upogebia deltaura* (Leach, 1815) |
| Animalia | Arthropoda | Malacostraca | Amphipoda | Urothoidae | *Urothoe* | *Urothoe elegans* | *Urothoe elegans* Spence Bate, 1857 |
| Animalia | Arthropoda | Malacostraca | Amphipoda | Urothoidae | *Urothoe* | *Urothoe grimaldii* | *Urothoe grimaldii* Chevreux, 1895 |
| Animalia | Arthropoda | Malacostraca | Amphipoda | Urothoidae | *Urothoe* | *Urothoe poseidonis* | *Urothoe poseidonis* Reibish, 1905 |
| Animalia | Arthropoda | Malacostraca | Amphipoda | Urothoidae | *Urothoe* | *Urothoe pulchella* | *Urothoe pulchella* (A. Costa, 1853) |
| Animalia | Mollusca | Bivalvia |  |  | *Varicorbula* | *Varicorbula gibba* | *Varicorbula gibba* (Olivi, 1792) |
| Animalia | Mollusca | Bivalvia | Venerida | Veneridae | *Venerupis* | *Venerupis corrugata* | *Venerupis corrugata* (Gmelin, 1791) |
| Animalia | Mollusca | Gastropoda | Littorinimorpha | Hydrobiidae | *Ecrobia* | *Ecrobia ventrosa* | *Ventrosia ventrosa* (Montagu, 1803) |
| Animalia | Mollusca | Bivalvia | Venerida | Veneridae | *Venus* | *Venus verrucosa* | *Venus verrucosa* Linnaeus, 1758 |
| Animalia | Arthropoda | Thecostraca | Verrucomorpha | Verrucidae | *Verruca* | *Verruca stroemia* | *Verruca stroemia* (O.F. Müller, 1776) |
| Animalia | Bryozoa | Gymnolaemata | Ctenostomatida | Victorellidae | *Victorella* | *Victorella muelleri* | *Victorella muelleri* (Kraepelin, 1887) |
| Animalia | Bryozoa | Gymnolaemata | Ctenostomatida | Victorellidae | *Victorella* | *Victorella pavida* | *Victorella pavida* Saville-Kent, 1870 |
| Animalia | Arthropoda | Malacostraca | Decapoda | Xanthidae | *Xantho* | *Xantho hydrophilus* | *Xantho hydrophilus* (Herbst, 1790) |
| Animalia | Arthropoda | Malacostraca | Decapoda | Xanthidae | *Xantho* | *Xantho pilipes* | *Xantho pilipes* A. Milne-Edwards, 1867 |
| Animalia | Arthropoda | Malacostraca | Decapoda | Xanthidae | *Xantho* | *Xantho poressa* | *Xantho poressa* (Olivi, 1792) |
| Animalia | Mollusca | Bivalvia | Mytilida | Modiolidae | *Xenostrobus* | *Xenostrobus securis* | *Xenostrobus securis* (Lamarck, 1819) |
| Animalia | Bryozoa | Gymnolaemata | Ctenostomatida | Vesiculariidae | *Zoobotryon* | *Zoobotryon verticillatum* | *Zoobotryon verticillatum* (Delle Chiaje, 1822) |
